# Systematic review of time lag between antibiotic use and rise of resistant pathogens among hospitalized adults in Europe

**DOI:** 10.1093/jacamr/dlad001

**Published:** 2023-01-20

**Authors:** Edith Poku, Katy Cooper, Anna Cantrell, Sue Harnan, Muna Abu Sin, Arina Zanuzdana, Alexandra Hoffmann

**Affiliations:** School of Health and Related Research (ScHARR), University of Sheffield, Sheffield, UK; School of Health and Related Research (ScHARR), University of Sheffield, Sheffield, UK; School of Health and Related Research (ScHARR), University of Sheffield, Sheffield, UK; School of Health and Related Research (ScHARR), University of Sheffield, Sheffield, UK; Department of Infectious Disease Epidemiology, Robert Koch Institute, Berlin, Germany; Department of Infectious Disease Epidemiology, Robert Koch Institute, Berlin, Germany; Department of Infectious Disease Epidemiology, Robert Koch Institute, Berlin, Germany

## Abstract

**Background:**

Antimicrobial resistance (AMR) causes substantial health and economic burden to individuals, healthcare systems and societies globally. Understanding the temporal relationship between antibiotic consumption and antibiotic resistance in hospitalized patients can better inform antibiotic stewardship activities and the time frame for their evaluation.

**Objectives:**

This systematic review examined the temporal relationship between antibiotic use and development of antibiotic resistance for 42 pre-defined antibiotic and pathogen combinations in hospitalized adults in Europe.

**Methods:**

Searches in MEDLINE, Embase, Cochrane Library and NIHR Centre for Reviews and Dissemination were undertaken from 2000 to August 2021. Pathogens of interest were *Escherichia coli*, *Klebsiella pneumoniae*, *Streptococcus pneumoniae*, *Staphylococcus aureus*, *Enterococcus faecium*, CoNS, *Pseudomonas aeruginosa* and *Acinetobacter baumannii* complex.

**Results:**

Twenty-eight ecological studies and one individual-level study were included. Ecological studies were predominantly retrospective in design (19 studies) and of reasonable (20 studies) to high (8 studies) methodological quality. Of the eight pathogens of interest, no relevant data were identified for *S*. *pneumoniae* and CoNS. Across all pathogens, the time-lag data from the 28 ecological studies showed a similar pattern, with the majority of studies reporting lags ranging from 0 to 6 months.

**Conclusions:**

Development of antibiotic resistance for the investigated antibiotic/pathogen combinations tends to occur over 0 to 6 months following exposure within European hospitals. This information could inform planning of antibiotic stewardship activities in hospital settings.

## Introduction

Antimicrobial resistance (AMR) is associated with substantial health and economic burden to individuals and healthcare systems.^1,2^ The WHO^3,4^ promotes antibiotic stewardship (ABS) programmes and activities in an effort to optimize the use of antibiotics and slow down the dramatic increasing trend in antibiotic resistance. Those efforts are supported by European institutions and initiatives, e.g. European Center for Disease Prevention and Control (ECDC), and addressed in national action plans to combat antibiotic resistance.^5^ For local ABS activities, robust surveillance data are needed about antibiotic use and antibiotic resistance in clinical settings as well as an integrated analysis of data from both, often independently implemented, surveillance systems. A tool for the integrated analysis of antibiotic consumption and resistance data was developed for hospitals in Germany in 2019 to support local ABS activities and programmes.^6^ One challenge in interpreting data in an integrative approach and mathematical modelling of AMR is the temporal relationship between antibiotic consumption (i.e. drug pressure) and the development of AMR.

Previous reviews^7–10^ have assessed the temporal relationship between antibiotic consumption and development of resistance in ambulatory and primary care settings. Generally, the reviews^7–9^ found evidence for an association between antibiotic consumption and the development of bacterial resistance, while findings on evidence for associations as well as time to emergence of resistance were not consistent for all antibiotics or bacteria. This may be explained by differences in review methodologies and scopes. Bell and colleagues^7^ included 243 studies (case–control, cross-sectional, ecological and experimental studies) across all antibiotics and bacteria.^7^ The time between consumption and resistance was 6 months or less in 53%, more than 6 months in 23%, and unclear in the remainder of the included studies.^7^ Costelloe *et al*.^8^ analysed 24 observational or experimental studies. Ecological studies that focused predominantly on the emergence of antibiotic resistance associated with urinary, respiratory or skin infections were excluded from this review.^8^ The review found that AMR developed shortly after antibiotic exposure (i.e. within 1 month) but gradually waned over time (up to 3 to 12 months).^8^ Bakhit and others,^9^ on the other hand, included 25 individual-level studies of varied study designs involving 1461 adults and 16 353 children and noted that resistance increased immediately after treatment and generally persisted for 1 to 3 months.

This current review was undertaken to provide better understanding of the body of evidence on the temporal relationship between antibiotic consumption and emergence of resistance in hospitalized patients. Temporal relationship is likely to vary between different countries with differing healthcare systems; one important consideration was the context (e.g. relating to treatment guidelines; infection prevention and control protocols; ABS practices, standards of AMR measurement; care setting and baseline resistance) of available evidence. For this reason, this review focuses on hospitals within Europe.

## Methods

The review examined the temporal relationship between antibiotic consumption and the development of antibiotic resistance. The review protocol was registered with the International Prospective Register of Systematic Reviews (PROSPERO) on 1 September 2021 and was last updated on 11 January 2022 (registration number CRD42021274957).

### Literature searches

Searches in MEDLINE, Embase, the Cochrane Library and archives of the NIHR Centre for Reviews and Dissemination (CRD) were undertaken in August 2021. The MEDLINE search strategy (File S1, available as Supplementary data at *JAC-AMR* Online) was adapted for use in other bibliographic databases. Search terms related to antibiotic resistance and antibiotic consumption or exposure. The search was conducted with and without additional terms for the hospital setting. All records from searches using hospital terms were checked in full. However, records from the broader search without hospital terms were considered as an extra data source and were screened using targeted keywords such as time-series, ARIMA, temporal, lag, cross-correlation, delay and dynamic transfer function. Based on previous reviews,^7–10^ a publication year limit from 2000 onwards was applied to reflect current trends of antibiotic resistance.

Supplementary searches using targeted keywords, as referred to earlier, were conducted in websites of international and national organizations including the ECDC, the Robert Koch Institute (RKI), the Surveillance Network France, Instituto de Salud Carlos III, WHO and the US CDC. Reference lists of included studies and relevant reviews were also examined to identify additional publications.

### Inclusion and exclusion criteria

Eligible study types were ecological studies and individual-level studies reporting on the temporal relationship between antibiotic use and subsequent emergence of antibiotic resistance for specific antibiotic and pathogen (drug/bug) combinations (See Files S2 and S3). Ecological studies generally reported the time lag that provided the best-fit correlation between time series for antibiotic consumption and resistance. Whereas individual studies reported time-lag data, which could consist of either ORs for resistance in patients with/without prior antibiotic exposure at different timepoints or the number of days to resistance development in individuals receiving antibiotics.

Eight pathogens were considered, based on the WHO priority pathogens list for research and development:^11^*Escherichia coli*, *Klebsiella pneumoniae*, *Streptococcus pneumoniae*, *Staphylococcus aureus*, *Enterococcus faecium*, Coagulase-negative Staphylococci (CoNS), *Pseudomonas aeruginosa* and *Acinetobacter baumannii* complex, with the potential to broaden to other species within the same genus in the event of limited data on a specific pathogen. The population of interest was hospitalized adults (or studies in mixed age groups), colonized or infected with pathogens of interest. Studies conducted within Europe, considered as the EU, European Economic Area (EEA) and the UK, were eligible for inclusion. Where limited data were identified for any pathogen, the potential to broaden the selection criteria to high- or middle-income countries outside the EU/EEA was considered. Studies published in English, German, French or Spanish from 2000 to August 2021 were included.

Exclusion criteria were as follows: studies with a publication date preceding 2000, coinfection with multiple pathogens, studies specific to children, the use of combination preparations of antibiotics, and studies conducted in low-income countries.

### Study selection

A two-staged selection of studies was conducted using pre-specified criteria. Two reviewers (E.P. or K.C.) checked titles and abstracts of retrieved records. One reviewer checked titles and abstracts of retrieved records. A second reviewer examined a 10% sample, early in the selection process. The two reviewers compared and discussed title and abstract decisions for the initial screening in order to improve consistency of subsequent study selection. The level of agreement between two reviewers during the initial selection process resulted in a kappa statistic of 0.84, indicating very good agreement. Disagreements were resolved by consensus following re-examination of the review protocol and feedback from the wider review team. The two reviewers discussed their understanding of the eligibility criteria at this stage to improve agreement in the next stages of the selection process. Subsequently, full-text articles of selected abstracts were then checked for eligibility. Any disagreements were resolved by discussion or referral to a third researcher when needed.

### Data extraction and quality assessment

Data were extracted into a pre-piloted Microsoft Excel^®^ form. Abstracted data included study characteristics, antibiotic susceptibility testing methods, antibiotic use and temporal relationship between antibiotic use and emergence of resistance. Data were checked by a second reviewer.

In the absence of an appropriate relevant quality assessment tool, bespoke criteria were applied to assess the methodological quality and relevance of included studies. Selected criteria were informed by the recommendations of the Quality Assessment Tool for Quantitative Studies set out by the Effective Public Health Practice Project (https://www.ephpp.ca/PDF/QADictionary_dec2009.pdf) and the quality appraisal approach reported by Costelloe and colleagues.^8^ For ecological studies, assessment of included studies focused on: (1) generalizability of findings to hospitalized adults within Europe; (2) reliability of quantifying antibiotic use; (3) reliability of reporting antibiotic resistance; (4) appropriateness of study design to estimate a temporal relationship (e.g. time lag) between antibiotic use and emergence of resistance; and (5) adjustment(s) for key confounders such as other antibiotic use and/or infection control measures. For studies with an individual-level study design, an additional item related to: (6) unbiased selection of a control group was assessed. Criteria were rated as ‘yes’, ‘no’ or ‘unclear’. Studies with five or more ‘yes’ responses were considered high quality; three to four ‘yes’ responses were considered to be reasonable quality and those with less than three ‘yes’ responses were noted as low quality. Details of applied criteria are outlined in File S4.

### Data synthesis

Data were summarized and presented in narrative and tabular summaries. Extensive clinical and methodological heterogeneity was noted in included ecological and individual-level studies. Time-lag outcomes reported in ecological studies were presented as discrete outcomes (e.g. lag of 1 month) or a range of outcomes, with limited or no information about uncertainty. For these reasons, meta-analysis was considered to be inappropriate.

## Results

**Figure 1.**
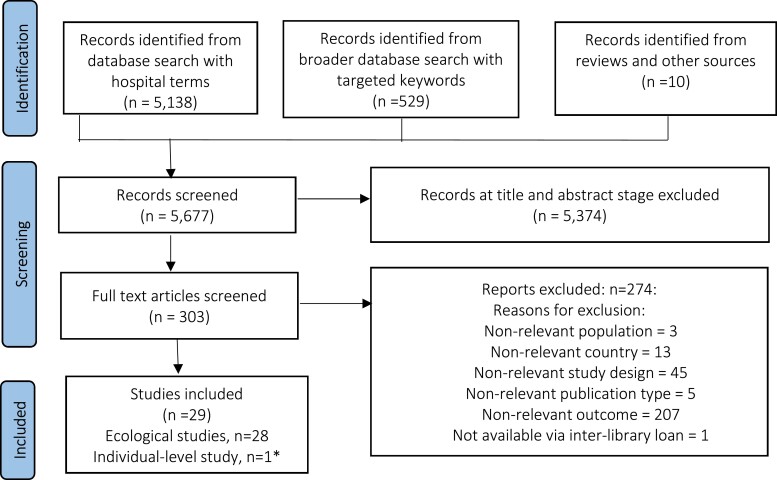
Flow diagram of study selection. *Summary of individual-level studies is presented in File S5.

Overall, 28 ecological^12–39^ and one case–control study^40^ were eligible for inclusion (Figure 1). The case–control study reported by Dualleh *et al*.^40^ assessed the effect of antibiotic use (0 to 6 months, 6 to 12 months or 12 to 24 months prior to enrolment) on colonization with extended-spectrum β-lactamase (ESBL)-producing Enterobacterales. Prior use of fluoroquinolones during all three time periods was associated with incidence of ESBL-producing Enterobacterales, while prior use of penicillins or macrolides showed mixed results.^40^ Details of the individual-level study^40^ are presented separately in File S5 because of the differences in study designs and the available data on time-lag outcomes compared with the 28 ecological studies. Therefore, the synopsis here focuses on the ecological studies.

### Study settings

Ecological studies were reported as retrospective (*n* = 19); prospective (*n* = 1) and unclear (*n* = 8) in terms of study design. Studies were conducted in Germany,^21,22,25,32,37^ France,^18,20,28,36,38^ Scotland,^24,29,30^ England,^39^ Northern Ireland,^12,13^ Romania,^15,19^ Switzerland,^34,35^ Greece,^23^ Hungary,^33^ Norway,^14^ Slovenia,^16^ Spain^27^ and Serbia,^31^ while a further study included data from four countries in Europe.^26^ Where reported, capacity ranged from 11 bed to 3500 bed hospital settings. The majority of studies included admissions on various medical and surgical wards, whereas three studies^15,17,31^ focused exclusively on isolates from individuals admitted to ICUs in a single hospital (Table 1).

**Table 1.** Study characteristics, ecological studies

Study details	Country	Study duration (years)	Healthcare setting (*n*)	Capacity of healthcare setting(s) (beds)	Schedule for resistance and consumption data collection	Unit for antibiotic consumption	Unit for resistance	Method(s) of susceptibility testing	Guidelines for testing
Aldeyab, 2008^13^	Northern Ireland	5	Hospital (1)	462	Monthly	DDD/100 BDs	Incidence/100 BDs	VITEK 2	CLSI (formerly NCCLS), version NR
Aldeyab, 2012^12^	Northern Ireland	5	Hospital (1)	411	Monthly	DDD/1000 BDs	Incidence/1000 BDs	VITEK	CLSI, version NR
Aldrin, 2013^14^	Norway	5–6	Hospitals (3), NR	NR	Monthly	DDD/100 BDs	Incidence/100 BDs	NR	NR
Baditoiu, 2017^15^	Romania	2	ICU (1)	27 (1100 in hospital)	Quarterly	DDD/1000 PDs	Incidence/1000 PDs	VITEK	CLSI, 2012
Beovic, 2011^16^	Slovenia	3	Hospital (1), 3 units^a^	NR	Monthly	DDD/100 PDs	Incidence/1000 PDs	Disc diffusion	CLSI, 2005
Berger, 2004^38^	France	3	ICU, medical and surgical wards (4 hospitals)	3500	Monthly	DDD/month	Incidence/month	MicroScan^®^, Dade Behring Inc., West Sacramento, CA, USA	NCCLS, 1998
Erdeljic, 2011^17^	Croatia	1.5	ICU (1)	11 (1700 in hospital)	Monthly	DDD/100 admissions	% Non-susceptible	Local standards, not specified	CLSI, 2005
Gallini, 2010^18^	France	4	Hospital (1)	2848	Monthly	DDD/1000 PDs	% Non-susceptible	VITEK 2 or disc diffusion	NCCLS, 1997
Gharbi, 2015^39^	England	10	Renal inpatients and dialysis units; hospital (1)	77	Yearly	DDD/100 occupied BDs/year	Incidence/100 000 occupied BDs/year	NR	NR
Ghenea, 2021^19^	Romania	2	ICU, medical and surgical ward (1 hospital)	NR	Monthly	DDD/100 PDs	Monthly resistance	VITEK 2; disc diffusion	NR
Hocquet, 2008^20^	France	6	Hospital (1)	NR	Monthly	DDD/1000 PDs	Incidence/1000 PDs	Disc diffusion	ACFSM, 2007
Kaier, 2009^21^	Germany	3	Hospital (1)	1600	Monthly	DDD/1000 PDs	Incidence/1000 PDs	Disc diffusion	NR
Kaier, 2009^22^	Germany	5	Hospital (1)	1600	Monthly	DDD/1000 PDs	Incidence/1000 PDs	NR	NR
Kritsotakis, 2008^23^	Greece	7	Hospital (1)	700	Bi-monthly	DDD/100 PDs	Incidence/1000 PDs	NR	NR
Lawes, 2015^24^	Scotland	16	Hospital (1), ICU and various wards^b^	1000	Monthly	DDD/1000 BDs	% CC22, CC30 and CC5 MRSA resistant strains	VITEK; disc diffusion; Epidemiological typing	CLSI, version NR (from inception); EUCAST (from 2012)
Lepper, 2002^25^	Germany	3	Hospital (1), major medical and surgical units	600	Monthly	DDD/month	% Resistance	VITEK; microbroth breakpoint dilution	German National Standard; NCCLS, 2000
Lopez-Lozano, 2019^26^	France, Hungary, Northern Ireland, Spain	6–25	Hospital (1)	1559	Monthly	DDD/1000 BDs	Incidence/10 000 occupied BDs	Disc diffusion, broth microdilution	EUCAST or CLSI, version NR
Lopez-Lozano, 2000^27^	Spain	8	Hospital (1)	400	Monthly	DDD/1000 PDs	% Non-susceptible	NR	NCCLS, 1993
Mahamat, 2005^28^	France	7	Hospital (1)	1659	Monthly	DDD/1000 PDs	% Resistant	VITEK 2; disc diffusion	ACFSM, 2000
Mahamat, 2007^29^	Scotland	8	Hospitals (2)	500	Monthly	DDD/1000 BDs	% Resistant	Disc diffusion	BSACWP criteria, 1991 (from study inception to 2001); CLSI, 2000 (for later part of study)
Monnet, 2004^30^	Scotland	5	Hospital (1)	1200	Monthly	DDD/1000 PDs	% Resistant	Disc diffusion	NR
Popovic, 2020^31^	Serbia	5	ICU (1)	12	Yearly	DDD/100 BDs	Incidence/1000 PDs	VITEK 2; disc diffusion	CLSI, version NR prior to 2017; EUCAST since 2017
Remschmidt, 2017^32^	Germany	2	Hospital (1), ICUs and various wards^c^	3000	Monthly	DDD/100 PDs	Incidence rate ratios	VITEK 2	EUCAST
Toth, 2019^33^	Hungary	13	Hospital (1)	1667	Monthly	DDD/100 BDs	Incidence/1000 occupied BDs	NR	NR
Vernaz, 2011^34^	Switzerland	8	Hospital (1)	2200	Monthly	DDD/1000 PDs	Incidence/1000 PDs	Disc diffusion	CLSI, 2009
Vernaz, 2008^35^	Switzerland	7	Hospital (1)	2200	Monthly	DDD/100 PDs	Incidence/100 PDs	NR	NR
Vibet, 2015^36^	France	7	Hospital (1)	3000	Monthly	DDD/1000 PDs	Incidence/1000 PDs	VITEK 2; combined disc test or modified combined synergy test	ACFSM, 2014
Willmann, 2013^37^	Germany	10	Hospital (1)	1513	Quarterly	DDD/1000 inpatient-days	% Resistant	VITEK 2; disc diffusion	CLSI, 2009

ACFSM, Antibiogram Committee of the French Society for Microbiology; BDs, bed days; BSACWP, British Society for Antimicrobial Chemotherapy Working Party; CLSI, Clinical and Laboratory Standards Institute; DDD, defined daily doses; EUCAST, European Committee on Antimicrobial Susceptibility Testing; ICU, Intensive Care Unit; NCCLS, National Committee for Clinical and Laboratory Standards; NR, not reported; PD, patient-days.

a Infectious diseases ward, abdominal surgery ward and surgical ICU.

b General surgical and medical wards.

c Surgical, medical and haemato-oncological wards.

### Infection control and ABS

Reporting of infection control policies and ABS practices varied across studies. Measures included limiting use of antibiotics,^24,31,39^ the use of alcohol hand rub,^13,21,22,24–26,29^ compliance audits,^21,22,26,29^ and screening of patients with resistant pathogens.^13,21,24,26,29,30,35,36,26,32^ Practices varied in a single study^26^ that included data from five study sites in France, Hungary, Spain and Northern Ireland. Thirteen ecological studies^12,14–20,27,28,33,37,38^ did not explicitly present information about measures to control AMR. In terms of changes in infection control or ABS practices during the study period, no information was presented in six studies,^14,38,18,20,37^ whereas nine studies^15–17,26,30,31,25,33,36^ reported no change in infection control practices, with one study^25^ stating, further, no change in ABS staff during the study period. Remaining studies reported restricting use of fluoroquinolones^12^ and carbapenems^25,39^ and regular or intensified use of alcohol wipes or hand rub^13,21,22^ (File S6).

### Reporting of antibiotic consumption and antibiotic resistance

Reporting of antibiotic consumption varied. In ecological studies, defined daily doses (DDD)/month,^25,38^ DDD/100 admissions,^17^ DDD/100 patient-days (PDs),^16,19,23,32,35^ DDD/1000 PDs,^15,18,20–22,27,28,30,34,36,37^ DDD/100 bed-days (BDs)^13,14,31,33,39^ and DDD/1000 BDs^24,26,29^ were reported. Most studies (86%; 24 studies^12,13,15–18,20,21,23–38^) reported the exclusion of duplicate isolates during susceptibility testing. This information was unclear in one study^19^ and absent in three studies.^14,22,39^ Antibiotic resistance across studies was presented as: incidence/100 BDs^13,14^ or 1000 BDs^12^ or 10 000 PDs;^26^ incidence/1000 PDs;^12,15,16,20–23,31,36^ incidence/100 000 occupied BDs/year;^39^ incidence/month;^38^ incidence rate ratios;^32^ and monthly resistance^19^ as well as percentage of non-susceptible isolates^17,18,27^ or resistant isolates/strains.^24,25, 28–30,37^ Most studies reported using methods such as the VITEK method and disc diffusion for testing resistance. Reporting on standards for judging resistance was varied. Many studies (16/19) applied the National Committee for Clinical Laboratory Standards (NCCLS), Clinical and Laboratory Standards Institute (CLSI) or European Committee on Antimicrobial Susceptibility Testing (EUCAST) standards, depending on the study period (see Table 1). For analysis of the temporal relationship between antibiotic consumption and emergence of antibiotic resistance, 23 studies recorded data monthly,^12–14,16–22, 24–30,32–34,36,38,39^ while others used bimonthly,^23^ quarterly^15,37^ or yearly^31,39^ data collection schedules.

### Study quality and relevance

Overall, all 28 ecological studies met most quality assessment items and were of reasonable (20 studies) to high quality (8 studies). A summary of methodological quality of ecological studies is presented in Table 2. Applied criteria are outlined in File S4.

**Table 2.** Methodological quality and relevance, ecological studies

Study	1. Relates to hospitalized adults within Europe?^a^	2. Antibiotic use: reliable measure?	3. Resistance: reliable measure?	4. Study design appropriate to estimate time lag?	5. Adjustment for key confounders?
Aldeyab, 2008^13^	Yes	Yes	Yes	Yes	Yes
Aldeyab, 2012^12^	Yes	Yes	Yes	Yes	No
Aldrin, 2013^14^	Yes	Yes	Unclear	Yes	No
Baditoiu, 2017^15^	Yes	Yes	Yes	Yes	No
Beovic, 2011^16^	Yes	Unclear	Yes	Yes	Unclear
Berger, 2004^38^	Yes	Yes	Yes	Yes	Yes
Erdeljic, 2011^17^	Yes	No	Yes	Yes	Unclear
Gallini, 2010^18^	Yes	Yes	Yes	Yes	Unclear
Gharbi, 2015^39^	Yes	Yes	Unclear	Yes	Unclear
Ghenea, 2021^19^	Yes	Yes	Unclear	Yes	Unclear
Hocquet, 2008^20^	Yes	Unclear	Yes	Yes	Unclear
Kaier, 2009^21^	Yes	Yes	Unclear	Yes	Yes
Kaier, 2009^22^	Yes	Yes	Unclear	Yes	Yes
Kritsotakis, 2008^23^	Yes	Yes	Unclear	Yes	Yes
Lawes, 2015^24^	Yes	Yes	Yes	Yes	Yes
Lepper, 2002^25^	Yes	No	Yes	Yes	Yes
Lopez-Lozano, 2019^26^	Yes	Unclear	Yes	Yes	Yes
Lopez-Lozano, 2000^27^	Yes	Yes	Unclear	Yes	No
Mahamat, 2005^28^	Yes	Yes	Yes	Yes	Yes
Mahamat, 2007^29^	Yes	Yes	Yes	Yes	Yes
Monnet, 2004^30^	Yes	Yes	Unclear	Yes	Yes
Popovic 2020^31^	Yes	Yes	Yes	Yes	Unclear
Remschmidt, 2017^32^	Yes	Yes	Yes	No	Yes
Toth, 2019^33^	Yes	Yes	Unclear	Yes	Yes
Vernaz, 2011^34^	Yes	Yes	Yes	Yes	Yes
Vernaz, 2008^35^	Yes	Yes	Unclear	Yes	Yes
Vibet, 2015^36^	Yes	Yes	Yes	Yes	Yes
Willmann, 2013^37^	Yes	Yes	Yes	Yes	Yes

Criteria:

1. Yes if antibiotic use and resistance measured in hospital setting **and** study in Europe **and** study of adults or mixed ages.

2. Yes, if obtained from centralized database or source **and** expressed as DDD per *N* BDs or PDs.

3. Yes, if method reported **and** guidelines reported **and** expressed as incidence per *N* BDs or PDs **or** % resistance.

4. Yes, if time-series analysis and/or cross-correlation and/or dynamic regression.

5. Yes, if multivariate analysis adjusted for other antibiotic use **and/or** infection control measures.

a Assumed a mixture of adults and children where setting was a general hospital and it was not stated that children were excluded.

Eight studies^13,24,28,29,34,36–38^ scored ‘yes’ for all quality assessment items, whilst a further 12 studies^13,15,18,21–23,26,30–33,35^ scored ‘yes’ for four out of five items and 8 studies^14,16,17,19,20,25,27,39^ scored ‘yes’ for three out of five items. Therefore, 8 studies were of high quality while the other 20 studies were of reasonable quality according to the applied quality criteria. The item relating to the generalizability of the findings scored well in all 28 studies, since all studies were based in Europe, and measured antibiotic use and resistance in a hospital setting. The item that scored ‘yes’ the least frequently related to adjustments made in the analysis; however, 16 studies scored well. Analyses were not adjusted for other antibiotic use and infection control measures in four studies.^12,14,16,27^ A further seven studies scored ‘unclear’ for this item.^16–20,31,39^ The item relating to the methods used to assess resistance was often poorly reported, with 10 studies^14,19,21–23,27,30,33,35,39^ scoring ‘unclear’ for this item. This was usually because the laboratory methods used to ascertain susceptibility were not reported or the breakpoints or standards used to interpret susceptibility were not reported (Table 2).

### Outcomes of interest

Relevant outcome data were available for six of the eight pathogens of interest: *E. coli*, *K. pneumoniae*, *S. aureus*, *E. faecium*, *P. aeruginosa* and *A. baumannii*. No relevant data were identified for *S*. *pneumoniae* and CoNS, either for Europe or for other high- or middle- income countries. For some pathogens only limited data could be identified. Therefore, broadening the eligibility criteria led to the inclusion of a study conducted in Serbia^31^ and studies relating to broader genera of pathogens, such as *Klebsiella* spp., Vancomycin-resistent Enterococci (VRE), *Acinetobacter* spp. and ESBL producers including Enterobacterales.

To assess the strength of the association between antibiotic use and antibiotic resistance, most ecological studies conducted time-series analyses for both antibiotic use and resistance, then used correlation or regression analyses to assess the strength of the relationship. The time lag that gave the strongest association was then reported. The majority of analyses were multivariate with adjustments for prior resistance levels;^14,15,21–23, 26,27,30,33^ community antibiotic use^13,12,32,34,35^ and simultaneous in-hospital antibiotic use.^21,23–26,28,30,32–37^ Analyses were also adjusted for: infection control procedures (not specified);^13^ use of alcohol-based hand rub;^13,21,22^ prior^30,32^ or current frequency of admitted or screened patients with resistant pathogens;^21,22,24^ bed occupancy^24^ and length of hospital stay.^24^ Time-lag data were generally reported where a statistically significant association existed between antibiotic use and resistance; however, a few studies reported time-lag data for non-significant associations, which were included in a best-fit multivariate model. Tables 3–6 summarize the findings reported in ecological studies; details are presented in File S6.

**Table 3.** Summary of results, number of studies and pathogens

*E. coli* (5 studies)		*Klebsiella* spp. (4 studies)		*P. aeruginosa* (10 studies)		*S. aureus*/MRSA (8 studies)		Enterococci (VRE) (2 studies)		*Acinetobacter* spp. (3 studies)		ESBL producers (3 studies)	
*N* studies	Time lag	*N* studies	Time lag	*N* studies	Time lag	*N* studies	Time lag	*N* studies	Time lag	*N* studies	Time lag	*N* studies	Time lag
4^18,26,28,34^	0–6 months	1^19^	1–3 months	6^14,17,25–27,33^	0–2 months	8^13,22,24,26,29,30,35,38^	0–7 months	1^32^	1 month	2^26,33^	1–4 months	1^12^	1–2 months
1^33^	1–12 months	1^33^	1–6 months	2^16,20^	0–6 months			1^23^	2–6 months	1^31^	1 year^a^	1^21^	1–3 months
		2^31,39^	0–1 year^a^	1^15^	0–1 quarters^b^							1^36^	1–5 months
				1^37^	0–2 quarters^b^								

a Data aggregated yearly.

b Data aggregated quarterly.

**Table 4.** Time lag between use and resistance: *E. coli* and *Klebsiella* spp.

Antibiotic use	*E. coli* Total: 5 studies^18,26,28,33,34^				*Klebsiella* spp.^a^ Total: 4 studies^19,31,33,39^			
	Resistance to/resistance mechanism				Resistance to			
	Cephalosporins (NS)	Cephalosporins 3 + 4G	ESBL production	Fluoroquinolones	Carbapenems	Cephalosporins (NS)	Fluoroquinolones	Polymyxins
Carbapenems					1–2 months^19^		2–3 months^19^	0 years^31^
					6 months^33^			
					0 years^31^			
					1 year^39^			
Cephalosporins (NS)	1–12 months^33^					1–6 months^33^		
Cephalosporins 1 + 2G					0 years^31^			0 years^31^
Cephalosporins 3 + 4G		0–3 months^34^	1 month^26^					0 years^31^
		4 months^26^						
Fluoroquinolones		1–5 months^34^	2 months^26^	2–4 months^18^	1 year^31^		2–3 months^19^	1 year^31^
		3 months^26^		4–6 months^28^				
Penicillins ± β-lactamase inhibitor		3 months^34^						1 year^31^
Polymyxins					0–1 years^31^			0 years^31^

1+2G, 1^st^ and 2^nd^ generation; 3+4G, 3^rd^ and 4^th^ generation; E.coli, Escherichia coli; ESBL, extended-spectrum β-lactamase; NS, not specified; spp., species.

a *Klebsiella* spp. includes *K. pneumoniae* (two studies^31,39^), *K. pneumoniae* and *K. oxytoca* (one study^33^), and *Klebsiella* spp. (one study^19^).

**Table 5.** Time lag between use and resistance: *P. aeruginosa*

Antibiotic use	*P. aeruginosa* Total: 10 studies^14–17,20,25–27,33,37^								
	Resistance to/resistance mechanism								
	Aminoglycosides	Carbapenems	Cephalosporins 3 + 4G	Fluoroquinolones	Penicillin + β-lactamase inhibitor	Overproduction of MexXY-OprM^a^	3/4-class MDR^b^ *P. aeruginosa*	CR^c^ or 3/4-class MDR^b^ P. aeruginosa	XDR^d^ *P. aeruginosa*
Aminoglycosides	1 month^26^					0–6 months^20^			
Carbapenems	0 quarters^37^	0 months^17^	0–1 months^25^	0 quarters^37^	0–1 months^25^	0–6 months^20^	1 quarter^37^		
		0–1 months^14,25^							
		1 month^27^							
		0–2 months^33^							
		1–6 months^16^							
		0–1 quarters^15^							
		1 quarter^37^							
Cephalosporins, 3 + 4G	0 quarters^37^	0–1 months^25^	0–1 months^25^	0 quarters^37^	0–1 months^25^		0–1 quarters^37^		0 quarters^37^
			2 months^17^						
			0–2 quarters^37^						
Cephalosporins, 2 + 3 + 4G						0–6 months^20^			
Fluoroquinolones	1 month^26^ 0–1 quarters^37^			1 month^17^		0–6 months^20^			
Penicillins + β-lactamase inhibitor		0–1 months^25^	0–1 months^25^		0 months^14^	0–5 months^20^		0 quarters^37^	
		1 month^16^	0 quarters^37^		0–1 months^25^			0–1 quarters^15^	

2+3+4G, 2^nd^ and 3^rd^ and 4^th^ generation; CR, combined resistance; MDR, multi-drug resistant; XDR, extensively drug-resistant.

a Overproduction of MexXY-OprM in *P. aeruginosa* leads to low-level resistance to aminoglycosides, fluoroquinolones and 4G cephalosporins.

b 3/4-class MDR *P. aeruginosa* = non-susceptible or resistant to three or four of the following agents: piperacillin/tazobactam, ceftazidime, meropenem, ciprofloxacin. Definition as stated by Willman 2013^37^ and is not a universal definition.

c Combined resistant *P. aeruginosa* = combined resistance (CR) to ≥3 of ceftazidime, antipseudomonal penicillins, fluoroquinolones, aminoglycosides.^37^

d XDR *P. aeruginosa* = XDR, i.e. resistant to at least one agent in all, or all but one or two, antimicrobial categories.

**Table 6.** Time lag between use and resistance: *S. aureus*, enterococci*, Acinetobacter* spp. and ESBL producers

Antibiotic use	*S. aureus*/MRSA^a^ Total: 8 studies^13,22,24,26,29,30,35,38^			Enterococci (VRE)^b^ Total: 2 studies^23,32^	*Acinetobacter* spp.^c^ Total: 3 studies^26,31,33^	ESBL producers^d^ Total: 3 studies^12,21,36^
	Resistance to/resistance mechanism			Resistance mechanism	Resistance to	Resistance mechanism
	Fluoroquinolones	Lincosamides	MRSA incidence	VRE incidence	Carbapenems	ESBL production
Carbapenems					1–4 months^33^	2 months^36^
					3 months^26^	
Cephalosporins, 1 + 2G			1 month^22^			1 month^36^
Cephalosporins, 3 + 4G	0 months^38^		1–4 months^22^			1–5 months^36^
			2 months^13^			3 months^21^
			3 months^26^			
			5 monts^24^			
			4–5 months^35^			
			4–7 months^30^			
Fluoroquinolones	0 months^24^		1 month^13,35^		1 month^26^	1 month^21^
	0–4 months^38^		2 months^29^		1 year^31^	1–2 months^12^
			3 months^26^			3 months^36^
			2–4 months^24^			
			4 months^22^			
			4–5 months^30^			
Glycopeptides				1 months^32^		
				2 months^23^		
Lincosamides		0 months^24^	2 months^22^			
Penicillins ± β-lactamase inhibitor	0–5 months^38^		1 month^13,26^	6 months^23^		1–5 months^36^
			3 months^35^			
			2–5 months^24^			

1+2+3+4G, 2^nd^ and 3^rd^ and 4^th^ generation; ESBL, extended-spectrum β-lactamase; MRSA, methicillin-resistant Staphylococcus aureus; spp., species; VRE, Vancomycin-resistant Enterococci.

a All but one study of *S. aureus* evaluated MRSA.

b Enterococci includes vancomycin-resistant *E. faecium* and *E. faecalis* (one study^32^) or all VRE except *E. gallinarum* or *E. casseliflavus* (one study^23^).

c *Acinetobacter* spp. includes *A. baumannii* (two studies^26,33^) and *Acinetobacter* spp. (one study^31^).

d ESBL producers includes *E. coli*, *K. pneumoniae*, *E. cloacae* (one study^36^); *E. coli*, *E. cloacae*, *Klebsiella*, *Acinetobacter*, *Citrobacter* (one study^21^) and ESBL-producing bacteria, not specified (one study^12^).

### Time-lag results by pathogen

#### E. coli

For *E. coli*, across all studies (Tables 3 and 4) the time lag from antibiotic use to development of resistance ranged from 0 to 6 months across four studies^18,26,28,34^ and 1 to 12 months in one study.^33^ Cephalosporin use was associated with cephalosporin resistance (lag, 1 to 12 months^33^ in one study and 0 to 4 months in two further studies^26,34^), and with ESBL production (lag, 1 month^26^). Fluoroquinolone use was associated with fluoroquinolone resistance (lag, 2 to 6 months^18,28^), with third- and fourth-generation (3 + 4G) cephalosporin resistance (lag, 1 to 5 months^26,34^) and with ESBL production (lag, 2 months^26^). Penicillin with β-lactamase inhibitor (penicillin + β-lactamase inhibitor) use was associated with 3 + 4G cephalosporin resistance (lag, 3 months^34^).

#### Klebsiella spp.

Four studies^19,31,33,39^ assessed time-lag data in *Klebsiella* spp. (Tables 3 and 4). Two studies^31,39^ focused on *K. pneumoniae* only, while one study^33^ included *K. pneumoniae* and *Klebsiella oxytoca*, and one study^19^ included multiple *Klebsiella* species. Across all studies, the time lag ranged from 1 to 3 months in one study^19^ and 1 to 6 months in another,^33^ while in the two studies that aggregated data yearly, the time lag was 0 to 1 years.^31,39^

Carbapenem use was associated with carbapenem resistance (lag, 1 to 6 months^19,33^ or within the same year^31^ or the previous year^39^), with fluoroquinolone resistance (lag, 2 to 3 months^19^) and with polymyxin resistance (within the same year^31^). Cephalosporin use was associated with cephalosporin resistance (lag, 1 to 6 months^33^) and with carbapenem and polymyxin resistance (in the same year^31^).

Fluoroquinolone use was associated with fluoroquinolone resistance (lag, 2 to 3 months^19^) and with carbapenem and polymyxin resistance (in the following year^31^). Penicillin + β-lactamase inhibitor use was associated with polymyxin resistance (in the following year^31^). Polymyxin use was associated with carbapenem resistance (in the same and the following year^31^) and with polymyxin resistance (in the same year^31^).

#### P. aeruginosa

Ten ecological studies^14–17,20,25–27,33,37^ related to *P. aeruginosa* reported time-lag data (Tables 3 and 5). Across all studies, the time lag ranged from 0 to 2 months across six studies,^14,17,25–27,33^ from 0 to 6 months across two further studies,^16,20^ and from 0 to 2 quarters in two studies that aggregated data quarterly.^15,37^

Penicillin use (with or without β-lactamase inhibitors) was associated with penicillin ± β-lactamase inhibitor resistance (lag, 0 to 1 months^14,25^), with carbapenem resistance (lag, 0 to 1 months^16,25^), with 3 + 4G cephalosporin resistance (lag, 0 to 1 months^25^ or in the same quarter^37^), with overproduction of MexXY-OprM (lag, 0 to 5 months^20^) and with incidence of MDR *P. aeruginosa* isolates (within 0 to 1 quarter^15,37^). Overproduction of MexXY-OprM in *P. aeruginosa* was stated to lead to low-level resistance to aminoglycosides, fluoroquinolones and 4G cephalosporins.^20^

The use of 3 + 4G cephalosporins was associated with 3 + 4G cephalosporin resistance (lag, 0 to 2 months^17,25^ or within 0 to 2 quarters^37^), with penicillin + β-lactamase inhibitor resistance (lag, 0 to 1 months^25^), with aminoglycoside resistance (in the same quarter^37^), with carbapenem resistance (lag, 0 to 1 months^25^), with fluoroquinolone resistance (in the same quarter^37^), with incidence of MDR *P. aeruginosa* (lag, 0 to 1 quarter^37^) and with XDR *P. aeruginosa* (within the same quarter^37^). Use of second-generation (2G), 3G and 4G (2 + 3 + 4G) cephalosporins was associated with overproduction of MexXY-OprM (lag, 0 to 6 months^20^).

Aminoglycoside use was associated with aminoglycoside resistance (lag, 1 month^26^) and with overproduction of MexXY-OprM (lag, 0 to 6 months^20^). Carbapenem use was associated with carbapenem resistance (lag, 0 to 6 months^14,16,17,25,27,33^ or in the same or the next quarter^15,37^), with aminoglycoside resistance (in the same quarter^37^), with 3 + 4G cephalosporin resistance (lag, 0 to 1 months^25^), with fluoroquinolone resistance (within the same quarter^37^), with penicillin + β-lactamase inhibitor resistance (lag, 0 to 1 months^25^), with overproduction of MexXY-OprM (lag, 0 to 6 months^20^) and with three and our class MDR *P*. *aeruginosa* (lag, 1 quarter^37^). Fluoroquinolone use was associated with fluoroquinolone resistance (lag, 1 month^17^), with aminoglycoside resistance (lag, 1 month^26^ or within 0 to 1 quarter^37^) and with overproduction of MexXY-OprM (lag, 0 to 6 months^20^).

#### S. aureus and MRSA

Eight studies^13,22,24,26,29,30,35,38^ assessed time-lag data in *S. aureus* (Tables 3 and 6). All but one^38^ focused on MRSA. Overall, the time lag ranged from 0 to 7 months across the eight studies.^13,22,24,26,29,30,35,38^ Use of first-generation (1G) and 2G (1 + 2G) cephalosporins was associated with MRSA incidence (lag, 1 month^22^), while 3 + 4G cephalosporin use was associated with MRSA incidence (lag, 1 to 7 months^13,22,24,26,30,35^) and with fluoroquinolone resistance (lag, 0 months^38^).

Fluoroquinolone use was associated with MRSA incidence (lag, 1 to 5 months^13,22,24,26,29,30,35^) and with fluoroquinolone resistance (lag, 0 to 4 months^24,38^). Lincosamide use was associated with MRSA incidence (lag, 2 months^22^) and lincosamide resistance (in the same month^24^). Penicillin + β-lactamase inhibitor use was associated with MRSA incidence (lag, 1 to 5 months^13,24,26,35^) and with fluoroquinolone resistance (lag, 0 to 5 months^38^).

#### Enterococci

Two studies assessed time-lag data in enterococci; both studies focused on VRE (Tables 3 and 6). One study^32^ included vancomycin-resistant *Enterococcus. faecium and Enterococcus faecalis*, while the other study^23^ included all VRE except *Enterococcus gallinarum* and *Enterococcus casseliflavus*. Across both studies, the time lag was 1 month in one study^32^ and 2 to 6 months in the other.^23^ Glycopeptide use was associated with VRE incidence (lag, 1 to 2 months^23,32^), while penicillin + β-lactamase inhibitor use was also associated with VRE incidence (lag, 6 months^23^).

#### Acinetobacter spp.

Two studies^26,33^ focused on *A. baumannii* while one study^31^ included multiple *Acinetobacter* spp. (Tables 3 and 6). Carbapenem use was associated with carbapenem resistance (lag, 1 to 4 months^26,33^) while fluoroquinolone use was associated with carbapenem resistance (lag, 1 month,^26^ or in the subsequent year in a study^31^ that aggregated data yearly).

#### ESBL-producing bacteria

Three ecological studies^12,21,36^ assessed time-lag data for combined groups of ESBL-producing bacteria (Tables 3 and 6). Studies were included in the review as they included some pathogens relevant to the inclusion criteria. One study^36^ included *E. coli*, *K. pneumoniae* and *E. cloacae*; one study^21^ included *E. coli*, *E. cloacae*, *Klebsiella*, *Acinetobacter* spp. and *Citrobacter* spp.; and the remaining study^12^ did not specify the pathogen types. Across all studies, the time lag was 1 to 2 months in one study,^12^ 1 to 3 months in another study^21^ and 1 to 5 months in a third study.^36^

Penicillin use (with or without β-lactamase inhibitors) was associated with ESBL production (lag, 1 to 5 months^36^). The use of 1 + 2G cephalosporins and 3 + 4G cephalosporins were associated with ESBL production (lag, 1 month^36^ and 3 to 5 months,^21,36^ respectively). ESBL production was also associated with carbapenem use (lag, 2 months^36^) and fluoroquinolone use (lag, 1 to 3 months^12,21,36^).

## Discussion

This review summarized evidence relating to the temporal relationship between antibiotic consumption and resistance for specific drug/bug combinations in eight pathogens (*E. coli*, *K. pneumoniae*, *S. pneumoniae*, *S. aureus*, *E. faecium*, CoNS, *P. aeruginosa* and *A. baumannii* complex) in hospitalized patients in the EU, the EEA and the UK. Broadening of the eligibility criteria to include studies in high- and middle-income countries was applied due to limited data; however, no relevant studies were identified for *S. pneumoniae* or CoNS. The time-lag data were mainly reported where there was a significant association between antibiotic exposure and antibiotic resistance. It is unclear whether time-lag data would be meaningful where there is no significant association. Therefore, the likelihood of publication bias was not considered in this review.

The most investigated pathogen was *P. aeruginosa* (10 studies) followed by *S. aureus*/MRSA (8 studies) and *E. coli* (5 studies). Across all pathogens, the time-lag data from the 28 ecological studies showed a similar pattern, with the majority of studies reporting time lags ranging from 0 to 6 months. In *E. coli* (5 studies), the time lag ranged from 0 to 6 months across four studies^18,26,28,34^ and 1 to 12 months in one study.^33^ In *Klebsiella* spp. (four studies), the time lag ranged from 1 to 3 months in one study^19^ and 1 to 6 months in another,^33^ while in two studies that aggregated data yearly, the time lag was 0 to 1 years.^31,39^ In *S. aureus* (8 studies), the time lag ranged from 0 to 7 months across the eight studies.^13,22,24,26,29,30,35,38^ In enterococci (two studies, both of VRE), the time lag was 1 month in one study^32^ and 2 to 6 months in the other.^23^ For *P. aeruginosa* (10 studies), the time lag ranged from 0 to 2 months across six studies^14,17,25–27,33^ and from 0 to 6 months across two further studies,^16,20^ while in two additional studies that aggregated data quarterly, the time lag was 0 to 1 quarter in one^15^ and 0 to 2 quarters in the other.^37^ In *Acinetobacter* spp. (three studies), the time lag ranged from 1 to 4 months in two studies^26,33^ and was 1 year in a further study that aggregated data yearly.^31^ In ESBL-producing pathogens (three studies), the time lag was 1 to 2 months in one study,^12^ 1 to 3 months in another study^21^ and 1 to 5 months in a third study.^36^

Ecological studies reported the time lag for the model with the best fit for assessing the association between antibiotic consumption and resistance and collected data monthly or quarterly. While most studies report time lags to appearance of antibiotic resistance of 0 to 6 months, one study reported time lags of 0 to 12 months.^33^ The range of 0 to 12 months includes the early appearance of resistance in the first 6 months after exposure to antibiotics and is therefore consistent with the findings of the majority of studies included in this review. We consider the reported time lags of up to 12 months in this study to reflect the length of persistence of antibiotic resistance once acquired. Two further studies^31,39^ report time lags of 12 months, but aggregated data yearly, which does not allow for assessing early appearance of resistance but supports the finding that antibiotic resistance (once acquired) can persist in the hospital setting for several months.

The findings of this review overall correspond to findings of two reviews conducted in the ambulatory setting. Costelloe *et al*.^8^ reviewed studies at the individual level and assessed the strength of associations of antibiotic consumption and resistance across different time periods.^8^ For *E. coli* from urinary tract infections, the strongest association was found for a time lag of 0 to 1 months, with a constant decrease of the strength of association with increasing time lags (0 to 3, 0 to 6 and 0 to 12 months, respectively). The analysis of pathogens of respiratory tract infections, including *S. pneumoniae*, in the reviews of Costelloe *et al*.^8^ and Bakhit *et al.*^9^ revealed an association of exposure to antibiotics and emergence of resistance within the first 3 months after exposure to various antibiotics. For *S. pneumoniae*, a pathogen causing community-acquired lower respiratory tract infections that usually do not require inpatient care, no relevant studies were identified in this review. It is worth noting that individual-level studies may find shorter time lags than ecological studies, which take into account spread of resistance within a population or setting. Overall, the current findings did not demonstrate substantial differences between classes of antibiotics or pathogens in terms of the outcome of interest.

Evidence relating to time lags for cross-resistance do not differ from those of concordant antibiotic classes. Overall, the review found that carbapenems and fluoroquinolones were most commonly associated with cross-resistance in a number of pathogens. Available literature supports the occurrence of cross-resistance following the use of carbapenems and fluoroquinolones in hospital settings.^41,42^ The review also found that cross resistance was reported most commonly for *P*. *aeruginosa* isolates. This may be explained by the volume of available relevant evidence. On the other hand, *P*. *aeruginosa* is known to be a major cause of hospital-acquired infections, which also possesses an inherent characteristic for the emergence of resistant strains, both for concordant and discordant antibiotic classes. Both characteristics may influence a propensity for cross-resistance due to antibiotic selection pressure. Overall, time lags for discordant antibiotic classes of antibiotic exposure and resistance did not appear to differ from those of concordant antibiotic classes, with both sets of time lags ranging from 0 to 6 months.

*P. aeruginosa*, *S. aureus* and appearance of MRSA are the most investigated pathogens within the studies included in this review. While resistance to fluoroquinolones and lincosamides within studies of *S. aureus* appears with a time lag of 0 to 5 months (time lag in two studies: 0 months in one study;^24^ 0 to 5 months in the other study^38^), resistance to methicillin/oxacillin (appearance of MRSA) tends to occur with a slightly greater time lag (range between 1 and 7 months in seven studies). This is surprising against the background that MRSA has the potential to be identified in colonized patients through screening measures and therefore should potentially be identified at an early stage. Hygiene measures such as isolation of patients at risk for MRSA or decolonization might explain this slightly later appearance of MRSA compared with resistance of fluoroquinolones and lincosamides in *S. aureus*. Overall, this difference in time lags should not be overinterpreted, since the different time lags derive from different studies applying different methodologies. Only the study by Lawes *et al.*^24^ investigated both resistance to fluoroquinolones in *S. aureus* and incidence of MRSA, with results supporting the trend described.

### Strengths and limitations

This review presents the most recent findings on the temporal relationship between antibiotic consumption and resistance for specific drug/bug combinations in European hospitals. Studies published since 2000 were identified to reflect data that are more relevant to current antibiotics, trends in resistance and settings. The search strategy led to the identification of an acceptable evidence base. Included studies were of reasonable to high methodological quality, although retrospective study designs were common. Most used standardized measures for data on antibiotic use and microbiological information and scored well on analytical methods.

There are a few limitations of this review. Firstly, it was not possible to assess the impact of potential confounders on time-lag outcomes, especially for studies that did not clearly present this information. For example, the review included studies conducted in diverse hospital departments where patients may have a range of comorbidities, and patient management including infection control and ABS measures adds to the complexity of different exposures and might have had an additional effect on resistance. The impact of these factors on selection pressure for resistance was not possible to elucidate, due to reported analyses. Secondly, due to study design and available data in the hospital setting, development of resistance was mostly analysed based on aggregated ecological data. Additionally, the potential effect of transmission could not be assessed systematically. The wider use of electronic health records may overcome some of these limitations and result in patient-based data collection and analyses for antibiotic use and subsequent resistance in the hospital setting.

Thirdly, the inclusion of studies reporting on pathogen genus instead of defined species may limit the generalizability of results. However, in the absence of data per species, this information could be a reasonable proxy for decision-making. Furthermore, the effect of 1G and 2G cephalosporins and different active substances of fluoroquinolones could not be assessed separately because included studies presented data of these antibiotics grouped as presented in this review. Fourthly, the available data did not permit analysis of the dose-effect and treatment duration on time-lag outcomes, nor in detail the time to resistance decay. Finally, study selection was completed by two reviewers with a robust checking of the study selection process. There is a risk of having missed relevant studies, which overall we consider to be minimal.

### Conclusions

The available evidence from ecological studies suggests that the development of antibiotic resistance for specific drug/bug combinations within a hospital population mainly occurs between 0 to 6 months after use of related antibiotics within European hospitals. Knowledge on the time lag for emergence of antibiotic resistance after antibiotic exposure for a set of comprehensive drug/bug combinations could help define time periods for monitoring and evaluation of ABS interventions and inform tools modelling the association of antibiotic exposure and resistance to support ABS activities in hospitals. Evidence on the time lag between reduction of antibiotic use and subsequent decline in resistant pathogens as a result of ABS is not reviewed yet and should be part of further reviews.

## Supplementary Material

dlad001_Supplementary_Data

## Supplementary data

Files S1 to S6 are available as Supplementary data at *JAC-AMR* Online.
