# Supplementary material for: Systematic review of time lag between antibiotic use and rise of resistant pathogens among hospitalized adults in Europe
Source: JAC Antimicrob Resist. 2023 Jan 20;5(1):dlad001. doi: 10.1093/jacamr/dlad001 (PMC9856344; doi:10.1093/jacamr/dlad001)
Supplement: dlad001_Supplementary_Data [file dlad001_supplementary_data.docx]

**SUPPLEMENTARY FILES**

Supplementary File 1: Medline Search Strategy

| Database: Ovid MEDLINE(R) and Epub Ahead of Print, In-Process, In-Data-Review & Other Non-Indexed Citations and Daily <1946 to August 23, 2021>  Search Strategy:  --------------------------------------------------------------------------------  1 drug resistance/ or drug resistance, microbial/ or exp drug resistance, bacterial/  2 (resistance or resistant).tw.  3 1 or 2  4 ("antibiotic consumption" or "antibiotic prescri*" or "antibiotic utili?ation" or "antibiotic use").tw.  5 "antibiotic exposure".tw.  6 (antibiotic consumption or antibiotic prescribing or antibiotic prescription or antibiotic usage or "antibiotic use" or antibiotic utilisation or antibiotic utilization or antibiotic utilization patterns).kw.  7 4 or 5 or 6  8 3 and 7  9 limit 8 to humans  10 limit 9 to yr="2000 -Current"  11 limit 10 to (english or french or german or spanish)  12 Hospitalization/  13 "hospital*".tw.  14 "inpatient*".tw.  15 "in-patient*".tw.  16 (hospitalisation or hospitalization).kw.  17 12 or 13 or 14 or 15 or 16  18 8 and 17  19 limit 18 to humans  20 limit 19 to yr="2000 -Current"  21 limit 20 to (english or french or german or spanish)  22 11 not 21 |
| --- |

Supplementary File 2: Study eligibility criteria

|  | **Inclusion** | **Exclusion** |
| --- | --- | --- |
| **Study designs** | 1) Ecological studies assessing antibiotic consumption and antibiotic resistance within a population (where these data are not connected at the individual level)  2) Individual-level studies assessing antibiotic resistance in individuals having received a course of antibiotics, analysed at the level of the individual  - Prospective and retrospective interventional or non-interventional study designs | - Studies assessing development of resistance in vitro, i.e. where the isolate is grown in culture and resistance assessed over time |
| **Population** | - Adults, or studies in mixed age groups | - Studies in children |
| **Pathogens of interest** | Infection or colonisation from one of the following pathogen:  - *Escherichia coli*  - *Klebsiella pneumoniae*  - *Streptococcus pneumoniae*  - *Staphylococcus aureus*- *Enterococcus faecium*  - Coagulase-negative *Staphylococci*  - *Pseudomonas aeruginosa*  - *Acinetobacter baumannii* complex  In the event of limited data on a specific pathogen, inclusion criteria may be broadened to other species within the same genus | - Other pathogens, not in scope  - Co-infection with multiple pathogens |
| **Antibiotics of interest** | - Specific antibiotic-pathogen (“drug-bug”) combinations (Table S2)  - Antibiotic monotherapy only | - Other drug-bug combinations, not in scope  - Multi-drug antibiotic treatment |
| **Setting and country** | Hospitals in  1) Europe (European Union, European Economic Area and UK)  2) Other high- and middle-income countries, in the event of limited EU or European data for a specific pathogen | - Community and primary care  - Low-income countries |
| **Outcomes** | Time-lag between antibiotic consumption and subsequent development of resistance in any of eight pathogens for relevant drug-bug combinations (Table S2)  Outcome formats included:  1) Reported time-lag outcomes; correlation coefficients of best fit from time-series (ecological study design);  2) Reported odds ratios for resistance in patients with/without prior antibiotic exposure (individual-level study design) | - Outcomes, not reporting the temporal relationship between antibiotic consumption and subsequent resistance of pathogens |
| **Language** | Studies written in English, German, French or Spanish | Studies written in other languages |
| **Publication types** | Full versions of primary studies | Abstracts, short reports, systematic reviews, editorials, case reports |
| **Publication dates** | Studies published in the past two decades (2000 to 2021) | Studies published earlier than the year 2000 |

Supplementary File 3: Antibiotic-pathogen ('drug-bug') combinations of interest

|  | ***E*. *coli*** | ***K*. *pneumoniae*** | ***S*. *pneumoniae*** | ***S*. *aureus*** | ***E*. *faecium*** | **Coagulase-negative *Staphylococci*** | ***P. aeruginosa*** | ***A. baumannii* complex** |
| --- | --- | --- | --- | --- | --- | --- | --- | --- |
| Penicillins |  |  |  | ✓ |  | ✓ | ✓ |  |
| Penicillin + inhibitor |  | ✓ |  |  | ✓ |  |  |  |
| Penicillin +/- inhibitor | ✓ | ✓ | ✓ |  |  |  |  |  |
| 2G Cephalosporin | ✓ | ✓ |  | ✓ |  | ✓ |  |  |
| 3G Cephalosporin | ✓ | ✓ | ✓ | ✓ |  | ✓ | ✓ |  |
| 3G Cephalosporin + inhibitor | ✓ | ✓ |  |  |  |  |  |  |
| 4G Cephalosporin | ✓ | ✓ |  |  |  |  | ✓ |  |
| Fluoroquinolones |  |  | ✓ | ✓ |  | ✓ | ✓ | ✓ |
| Carbapenems |  |  |  | ✓ |  | ✓ | ✓ | ✓ |
| Lincosamides |  |  |  | ✓ |  | ✓ |  |  |
| Aminoglycosides |  |  |  |  | ✓ |  |  |  |
| Glycopeptides |  |  |  | ✓ | ✓ |  |  |  |
| Macrolides |  |  | ✓ |  |  |  |  |  |
| Phosphonic acids | ✓ |  |  |  |  |  |  |  |
| Trimethoprim +/-sulphonamide |  |  |  |  |  |  |  | ✓ |
| ‘✓’ indicates the relevant antibiotic-pathogen (“drug-bug”) combination for time-lag outcome of interest  ‘+/-‘ indicates with or without  **Abbreviations**: *A. baumannii* complex, *Acinetobacter baumannii* complex; *E*. *faecium*, *Enterococcus faecium*; E. coli, *Escherichia coli*; *K*. *pneumoniae*, *Klebsiella pneumoniae*; *P*. *aeruginosa*, *Pseudomonas aeruginosa*; *S*. *aureus*, *Staphylococcus aureus*; *S*. *pneumoniae*, *Streptococcus pneumoniae*; 2G, second generation; 3G, third generation; 4G, fourth generation | | | | | | | | |

Supplementary File 4: Criteria for assessing study quality and relevance

| **Study quality item** | **Rating** | **Criteria** |
| --- | --- | --- |
| Ecological study design | | |
| 1. Relates to hospitalised adults within Europe? | Yes | Antibiotic use and resistance measured in hospital setting **and** study in Europe **and** study of adults or mixed ages |
|  | No | Does not meet criteria above |
|  | Unclear | Insufficient information **and/or** limited reporting to rate as ‘yes’ or ‘no’ |
| 2. Antibiotic use: reliable measure? | Yes | Information obtained from centralised database or source **and** expressed as DDD per N bed-days or patient-days |
|  | No | Does not meet criteria above |
|  | Unclear | Insufficient information **and/or** limited reporting to rate as ‘yes’ or ‘no’ |
| 3. Resistance: reliable measure? | Yes | Method reported **and** guidelines reported **and** expressed as incidence per N bed-days or patient-days or % resistance |
|  | No | Does not meet criteria above |
|  | Unclear | Insufficient information **and/or** limited reporting to rate as ‘yes’ or ‘no’ |
| 4. Study design appropriate to estimate time-lag? | Yes | Time-lag data obtained from time series analysis **and/or** cross-correlation and/or dynamic regression |
|  | No | Does not meet criteria above |
|  | Unclear | Insufficient information **and/or** limited reporting to rate as ‘yes’ or ‘no’ |
| 5. Adjustment for key confounders? | Yes | Multivariate analysis adjusted for other antibiotic use **and/or** infection control measures |
|  | No | Does not meet criteria above |
|  | Unclear | Insufficient information **and/or** limited reporting to rate as ‘yes’ or ‘no’ |
| Individual-level study design | | |
| 1. Relates to hospitalised adults within Europe? | Yes | Antibiotic use and resistance measured in hospital setting **and** study in Europe **and** study of adults or mixed ages |
|  | No | Does not meet criteria above |
|  | Unclear | Insufficient information **and/or** limited reporting to rate as ‘yes’ or ‘no’ |
| 2. Antibiotic use: reliable measure? | Yes | Information obtained from centralised database or source **and** expressed as DDD |
|  | No | Does not meet criteria above |
|  | Unclear | Insufficient information **and/or** limited reporting to rate as ‘yes’ or ‘no’ |
| 3. Resistance: reliable measure? | Yes | Method reported **and** guidelines reported |
|  | No | Does not meet criteria above |
|  | Unclear | Insufficient information **and/or** limited reporting to rate as ‘yes’ or ‘no’ |
| 4. Study design appropriate to estimate time-lag? | Yes | Reports suitable data on time from antibiotic use to resistance development or measurement |
|  | No | Does not meet criteria above |
|  | Unclear | Insufficient information **and/or** limited reporting to rate as ‘yes’ or ‘no’ |
| 5. Adjustment for key confounders? | Yes | Multivariate analysis adjusted for age **and/or** co-morbidities |
|  | No | Does not meet criteria above |
|  | Unclear | Insufficient information **and/or** limited reporting to rate as ‘yes’ or ‘no’ |
| 6. Unbiased selection of a control group? | Yes | Clearly defines the group not exposed to antibiotics and the group without resistance |
|  | No | Does not meet criteria above |
|  | Unclear | Insufficient information **and/or** limited reporting to rate as ‘yes’ or ‘no’ |
| Abbreviations: DDD, daily defined dose; N, number  Summary of methodological quality assessment:  Studies with five or more ‘yes’ responses = high quality;  Studies with three to four ‘yes’ responses = reasonable quality  Studies with less than three ‘yes’ responses = low quality. | | |

Supplementary File 5: Summary of included individual-level study, Dualleh et al. ^40^

**Study characteristics**

A prospective case-control study^40^ conducted in Denmark from January to April 2018 was included in the review. The study^40^ assessed ESBL-enterococci (ESBL-E) colonisation and prior antibiotic exposure (to penicillins, macrolides and fluoroquinolones, at different doses, at 0 to 6 months prior, 6 to 12 months prior and 12 to 24 months prior). Analysis was based on data from a larger multicentre national study (AntiBiotic Resistance in Emergency departments in Denmark, AB-RED study^43^) examining the frequency of colonisation with multi-drug resistant (MDR) pathogens (based on EUCAST criteria) in acutely admitted patients as well as information for prior antibiotic consumption (reported as DDDs/ person-year) from a national register.^40^ Cases were patients with ESBL-E colonisation while controls were those with no ESBL-E. The authors noted that in-hospital antibiotic consumption was greater in cases than controls (proportions not reported, p=0.001). Measures for infection control and ABS were not reported.^40^ However, the overall methodological quality of the study was considered to be high. Table S1 and Table S2 summarise the study characteristics and the methodological quality of the study.

**Time-lag results**

Reported odds ratios (ORs) based on multivariate logistic regression and adjusted for age, gender, body region and Charlson comorbidity index (CCI) indicated higher antibiotic use in ESBL-E cases compared with controls. No clear difference in ORs for prior antibiotic exposure at different time points were noted (data not presented) when the model included a reference group of patients who had not received the index antibiotic. However, when the reference group were patients without no antibiotic exposure ((Table S3), a relatively consistent and statistically significant trend of detection of ESBL-E cases was reported with exposure to penicillins or fluoroquinolones of at least 10 daily defined doses per person-years, at all time points.^40^ In comparison, reported ORs did not show a similar trend in relation to prior exposure or quantity of macrolides.^40^

**Table S1: Study characteristics, individual-level studies**

| **Study details**  **Design**  **Country**  **Study period** | **Healthcare setting (n),** | **Study population, n**  **Age, median (IQR), years** | **Antibiotic consumption**  **Unit**  **Measures for infection control and ABS** | **Method(s) of susceptibility testing**  **Guidelines for testing** |
| --- | --- | --- | --- | --- |
| Dualleh 2020^40^  Prospective, case control study^a^  Denmark  2018 | Emergency department, 8 Hospitals | **Cases:** Patients colonised with ESBL-E, n=216    **Controls:** Patients with no ESBL-E, n=up to 4763^b^ (controls)  Age: 68 (54 to 77) | Previous antibiotics in the 2 years before study entry (hospital or community)  DDDs/ PY  NR | ESBL bi-agar  EUCAST |
| ^a^ Data obtained from a larger multicentre national study (AntiBiotic Resistance in Emergency departments in Denmark, AB-RED study)  ^b^Number differed based on analysis reported.  **Abbreviations**: ABS, antibiotic stewardship; DDD/PY, defined daily dose per person-year; ESBL; Extended Spectrum beta-Lactamase; ESBL-E; ESBL-producing Enterobacterales; EUCAST, European Committee on Antimicrobial Susceptibility Testing; NR, not reported | | | | |

**Table S2: Methodological quality and relevance, individual-level study**

| **Study** | **1. Relates to hospitalised adults within Europe?*** | **2. Antibiotic use: reliable measure?** | **3. Resistance: reliable measure?** | **4. Study design appropriate to estimate time-lag?** | **5. Adjustment for key confounders?** | **6. Unbiased control group selection?**** |
| --- | --- | --- | --- | --- | --- | --- |
| Dualleh 2020^40^ | Yes | Yes | Yes | Yes | Yes | Yes |
| * assumed a mixture of adults and children where setting was a general hospital and it was not stated that children were excluded  **individual-level studies with control group only  **Criteria:**  1. Yes if resistance measured in hospital setting **and** study in Europe **and** study of adults or mixed ages  2. Yes, if obtained from centralised database **or** patient records **and** expressed as DDD  3. Yes, if method reported and guidelines reported  4. Yes, if reports clear data on time from antibiotic use to resistance development or measurement  5. Yes, if multivariate analysis adjusted for age **and/or** co-morbidities  6. Yes, if clearly defines the group not exposed to antibiotics and the group without resistance | | | | | | |

**Table S3: Time-lag results, case-control study (ESBL-producing bacteria)**

| **Study** | **Antibiotic use** | **Dose: DDD/PY** | **Antibiotics: 0-6 months prior** | | | | | | **Antibiotics: 6-12 months prior** | | | | | | **Antibiotics: 12-24 months prior** | | | | | |
| --- | --- | --- | --- | --- | --- | --- | --- | --- | --- | --- | --- | --- | --- | --- | --- | --- | --- | --- | --- | --- |
|  |  |  | **Cases: ESBL-E** | | **Controls: No ESBL-E** | | **OR^a^**  **(95% CI)** | **p-value** | **Cases: ESBL-E** | | **Controls: No ESBL-E** | | **OR^a^**  **(95% CI)** | **p-value** | **Cases: ESBL-E** | | **Controls: No ESBL-E** | | **OR^a^**  **(95% CI)** | **p-value** |
|  |  |  | **Abx** | **No Abx** | **Abx** | **No Abx** |  |  | **Abx** | **No Abx** | **Abx** | **No Abx** |  |  | **Abx** | **No Abx** | **Abx** | **No Abx** |  |  |
| Dualleh 2020^40^ | Penicillins | >0-10 | 18 | 98 | 391 | 2750 | 1.35 (0.81-2.25) | 0.208 | 18 | 60 | 492 | 1782 | 1.10 (0.65-1.89) | 0.718 | 15 | 74 | 479 | 2215 | 0.97  (0. 56-1.71) | 0.927 |
|  |  | >10-20 | 43 | 98 | 726 | 2750 | **1.73 (1.19-2.51)** | **0.004** | 47 | 60 | 826 | 1782 | **1.74 (1.18-2.57)** | **0.005** | 43 | 74 | 772 | 2215 | **1.72 (1.17-2.54)** | **0.006** |
|  |  | >20 | 36 | 98 | 568 | 2750 | **1.90 (1.26-2.87)** | **0.002** | 69 | 60 | 1271 | 1782 | **1.66 (1.15-2.39)** | **0.007** | 60 | 74 | 938 | 2215 | **2.07 (1.42-3.00)** | **<0.001** |
|  | Macrolides | >0-10 | 7 | 98 | 88 | 2750 | **2.34 (1.06-5.19)** | **0.037** | 10 | 60 | 166 | 1782 | 1.89 (0.93-3.84) | 0.080 | 9 | 74 | 130 | 2215 | **2.21 (1.09-4.50)** | **0.029** |
|  |  | >10-14 | 10 | 98 | 171 | 2750 | 1.80 (0.91-3.55) | 0.090 | 15 | 60 | 265 | 1782 | **1.84 (1.03-3.27)** | **0.038** | 12 | 74 | 213 | 2215 | 1.82 (0.96-3.45) | 0.068 |
|  |  | >14 | 9 | 98 | 140 | 2750 | 2.01 (0.98-4.13) | 0.057 | 22 | 60 | 319 | 1782 | **2.20 (1.32-3.66)** | **00.002** | 15 | 74 | 227 | 2215 | **2.19 (1.22-3.99)** | **0.010** |
|  | FLQ | >0-10 | NR^b^ | 98 | 62 | 2750 | 1.88 (0.69-5.10) | 0.217 | 7 | 60 | 97 | 1782 | **2.35 (1.02-5.42)** | 0.045 | NR^b^ | 74 | 84 | 2215 | 1.14 (0.34-3.75) | 0.835 |
|  |  | >10-20 | 11 | 98 | 106 | 2750 | **3.37 (1.73-6.59)** | **<0.001** | 17 | 60 | 188 | 1782 | **3.16 (1.79-5.60)** | **<0.001** | 15 | 74 | 156 | 2215 | **3.17 (1.74-5.81)** | **<0.001** |
|  |  | >20 | NR^b^ | 98 | 26 | 2750 | **6.56 (2.46-17.46)** | **<0.001** | 8 | 60 | 68 | 1782 | **4.34 (1.87-10.06)** | **0.001** | 11 | 74 | 45 | 2215 | **8.74 (4.20-18.21)** | **<0.001** |
| ^a^OR = (odds of index antibiotic exposure vs. no antibiotic exposure for cases) vs. (odds of index antibiotic exposure vs. no antibiotic exposure for controls).  **Bold** = statistically significant at 5% level.  ^b^Not reported (NR) number where <5 observations.  **Abbreviations:** Abx, antibiotics; CI, confidence interval; DDD/PY, defined daily dose per person-year; ESBL, extended-spectrum beta-lactamase; ESBL-E, ESBL-producing *Enterobacterales*; FLQ, fluoroquinolones | | | | | | | | | | | | | | | | | | | | |

Supplementary File 6: Results - specific antibiotic-pathogen combinations (ecological studies)

**Time-lag results: E. coli (ecological study design)**

| **Study** | **Pathogen** | **Antibiotic use** | **Resistance to/ Resistance mechanism** | **Statistics** | **Model** | **Lag (mo)^a^** | **Coefficient** | | | | | | **R^2^** | **Adjusted variables** |
| --- | --- | --- | --- | --- | --- | --- | --- | --- | --- | --- | --- | --- | --- | --- |
|  |  |  |  |  |  |  | **Type** | **Coeff** | **SE** | **T-ratio** | **95% CI** | **p-value** |  |  |
| ***Escherichia coli*** | | | | | | | | | | | | | | |
| Toth 2019^33^ | *E. coli* | Cephalosporins | Cephalosporins | DR | NR | 1 | NR | 0.017 | NR | NR | 0.010 to 0.024 | <0.001 | NR | Prior resistance |
| Toth 2019^33^ | *E. coli* | Cephalosporins | Cephalosporins | DR | NR | 4 | NR | 0.01 | NR | NR | 0.003 to 0.017 | 0.007 | NR | Prior resistance |
| Toth 2019^33^ | *E. coli* | Cephalosporins | Cephalosporins | VAR | BV | 3-12 | HMSRRH | 0.17 | NR | NR | 0.065 to 0.231 | NR | NR | Prior resistance |
| Toth 2019^33^ | *E. coli* | Cephalosporins | Cephalosporins | VAR | MV | 2-12 | HMSRRH | 0.146 | NR | NR | 0.040 to 0.232 | NR | NR | Prior resistance; other Abx |
| Vernaz 2011^34^ | *E. coli* | Cephalosporins 3G (ceftriaxone) | Cephalosporins 4G (cefepime) | TSA, ARIMA, LTF | MV | 0 | NR | 0.0041 | 0.0017 | 2.4047 | NR | 0.0195 | 0.51 | Prior resistance; other Abx (hosp+comm) |
| Vernaz 2011^34^ | *E. coli* | Cephalosporins 4G (cefepime) | Cephalosporins 4G (cefepime) | TSA, ARIMA, LTF | MV | 3 | NR | 0.0034 | 0.0016 | 2.1502 | NR | 0.0358 | 0.51 | Prior resistance; other Abx (hosp+comm) |
| Lopez-Lozano 2019^26^ | *E. coli* | Cephalosporins 3+4G | Cephalosporins 4G (cefepime) | TSA+MV | MV | 4 | NR | 0.197 | NR | 4.1 | 0.103 to 0.291 | <0.0001 | 0.301 | Prior resistance; other Abx |
| Lopez-Lozano 2019^26^ | *E. coli* | Cephalosporins 3G | ESBL production | TSA+MV | MV | 1 | NR | 0.061 | NR | 3.28 | 0.029 to 0.093 | 0.0012 | 0.622 | Prior resistance; other Abx |
| Lopez-Lozano 2019^26^ | *E. coli* | Fluoroquinolones | Cephalosporins 4G (cefepime) | TSA+MV | MV | 3 | NR | 0.105 | NR | 2.2 | 0.012 to 0.198 | 0.0272 | 0.301 | Prior resistance; other Abx |
| Vernaz 2011^34^ | *E. coli* | Fluoroquinolones (ciprofloxacin) | Cephalosporins 4G (cefepime) | TSA, ARIMA, LTF | MV | 1 | NR | 0.0043 | 0.0013 | 3.2126 | NR | 0.0022 | 0.51 | Prior resistance; other Abx (hosp+comm) |
| Vernaz 2011^34^ | *E. coli* | Fluoroquinolones (ciprofloxacin) | Cephalosporins 4G (cefepime) | TSA, ARIMA, LTF | MV | 5 | NR | 0.0045 | 0.0014 | 3.2045 | NR | 0.0022 | 0.51 | Prior resistance; other Abx (hosp+comm) |
| Lopez-Lozano 2019^26^ | *E. coli* | Fluoroquinolones | ESBL production | TSA+MV | MV | 2 | NR | 0.122^b^ -0.269 0.010 | NR | 6.07 -2.34 0.06 | 0.085 to 0.159 -0.495 to -0.044 -0.300 to 0.319 | <0.0001 0.0203 0.9522 | 0.622 | Prior resistance; other Abx |
| Gallini 2010^18^ | *E. coli* | Fluoroquinolones (ciprofloxacin) | Fluoroquinolones (ciprofloxacin) | TSA, ARIMA, LTF, DR | MV | 4 | NR | 0.13 | 0.04 | NR | NR | 0.0006 | 0.465 | Prior resistance; other Abx (fluoroquinolones) |
| Mahamat 2005^28^ | *E. coli* | Fluoroquinolones (ciprofloxacin) | Fluoroquinolones (ciprofloxacin) | TSA, ARIMA, LTF, DR | MV | 4 | NR | 0.73 | 0.18 | 4.14 | NR | <0.001 | 0.4 | Other Abx (quinolones) |
| Mahamat 2005^28^ | *E. coli* | Fluoroquinolones (ciprofloxacin) | Fluoroquinolones (ofloxacin) | TSA, ARIMA, LTF, DR | MV | 4 | NR | 0.65 | 0.24 | 2.73 | NR | <0.01 | 0.64 | Other Abx (quinolones) |
| Gallini 2010^18^ | *E. coli* | Fluoroquinolones (levofloxacin) | Fluoroquinolones (ciprofloxacin) | TSA, ARIMA, LTF, DR | MV | 2 | NR | 0.22 | 0.04 | NR | NR | <0.0001 | 0.465 | Prior resistance; other Abx (fluoroquinolones) |
| Mahamat 2005^28^ | *E. coli* | Fluoroquinolones (norfloxacin) | Fluoroquinolones (ciprofloxacin) | TSA, ARIMA, LTF, DR | MV | 5 | NR | 0.63 | 0.3 | 2.07 | NR | <0.05 | 0.4 | Other Abx (quinolones) |
| Mahamat 2005^28^ | *E. coli* | Fluoroquinolones (norfloxacin) | Fluoroquinolones (ofloxacin) | TSA, ARIMA, LTF, DR | MV | 6 | NR | 0.53 | 0.14 | 3.88 | NR | <0.001 | 0.64 | Other Abx (quinolones) |
| Gallini 2010^18^ | *E. coli* | Fluoroquinolones (ofloxacin) | Fluoroquinolones (ciprofloxacin) | TSA, ARIMA, LTF, DR | MV | 2 | NR | 0.19 | 0.03 | NR | NR | <0.0001 | 0.465 | Prior resistance; other Abx (fluoroquinolones) |
| Mahamat 2005^28^ | *E. coli* | Fluoroquinolones (ofloxacin) | Fluoroquinolones (ciprofloxacin) | TSA, ARIMA, LTF, DR | MV | 4 | NR | 0.82 | 0.08 | 10.51 | NR | <0.0001 | 0.4 | Other Abx (quinolones) |
| Mahamat 2005^28^ | *E. coli* | Fluoroquinolones (ofloxacin) | Fluoroquinolones (ofloxacin) | TSA, ARIMA, LTF, DR | MV | 4 | NR | 0.81 | 0.09 | 9.45 | NR | <0.0001 | 0.64 | Other Abx (quinolones) |
| Vernaz 2011^34^ | *E. coli* | Penicillin/B-lact inhib (piperacillin/ tazobactam) | Cephalosporins 4G (cefepime) | TSA, ARIMA, LTF | MV | 3 | NR | 0.0099 | 0.0042 | 2.3073 | NR | 0.0247 | 0.51 | Prior resistance; other Abx (hosp+comm) |
| ^a^Lag in months unless otherwise stated.  ^b^Different coefficients for different Abx dose ranges.  **Abbreviations:** 1/2/3/4G, 1^st^/2^nd^/3^rd^/4^th^ generation; Abx, antibiotics; ARIMA, autoregressive integrated moving average; B-lact inhib, B-lactamase inhibitor; BV, bivariate; CCA, Cross correlation analysis; CI, confidence interval; coeff, coefficient; comm, community; DR, dynamic regression; *E. coli, Escherichia coli*; ESBL, extended spectrum beta-lactamase; HMSRRH, highest magnitude of significant response in the response horizon; hosp, hospital; LTF, linear transfer function; mo, months; MV, multivariate; MVA, multivariate analysis; MVR, multivariate regression; prev, prevalence; qtr, quarters; RC, regression coefficient; regr, regression; SE, standard error; spp, species; TSA, time series analysis; UV, univariate; VAR, vector autoregressive model; yr, years. | | | | | | | | | | | | | | |

**Time-lag results: K. pneumoniae and Klebsiella spp (ecological study design)**

| **Study** | **Pathogen** | **Antibiotic use** | **Resistance to/ Resistance mechanism** | **Statistics** | **Model** | **Lag (mo)^a^** | **Coefficient** | | | | | | **R^2^** | **Adjusted variables** |
| --- | --- | --- | --- | --- | --- | --- | --- | --- | --- | --- | --- | --- | --- | --- |
|  |  |  |  |  |  |  | **Type** | **Coeff** | **SE** | **T-ratio** | **95% CI** | **p-value** |  |  |
| ***Klebsiella pneumoniae* and *Klebsiella* spp** | | | | | | | | | | | | | | |
| Popovic 2020^31^ | *K. pneumoniae* | Carbapenems | Carbapenems (doripenem) | CCA | UV | 0 yr | CCC | NR | NR | NR | NR | NR | 0.65 | NR |
| Popovic 2020^31^ | *K. pneumoniae* | Carbapenems | Carbapenems (ertapenem) | CCA | UV | 0 yr | CCC | NR | NR | NR | NR | NR | 0.5 | NR |
| Toth 2019^33^ | *K. pneumoniae;*  *K. oxytoca* | Carbapenems | Carbapenems | DR | NR | 6 | NR | 0.005 | NR | NR | 0.003 to 0.006 | <0.001 | NR | Prior resistance |
| Ghenea 2021^19^ | *Klebsiella spp* | Carbapenems (imipenem) | Carbapenems (meropenem) | TSA, regr | NR | 1 | NR | 0.0243 | NR | NR | −0.0043 to 0.0529 | 0.078 | 0.627 | Other Abx (carbapenems) |
| Ghenea 2021^19^ | *Klebsiella spp* | Carbapenems (imipenem) | Carbapenems (meropenem) | TSA, regr | NR | 2 | NR | 0.0075 | NR | NR | 0.0011 to 0.0130 | 0.032 | 0.753 | Other Abx (carbapenems) |
| Ghenea 2021^19^ | *Klebsiella spp* | Carbapenems (meropenem) | Carbapenems (meropenem) | TSA, regr | NR | 1 | NR | −0.0016 | NR | NR | −0.0057 to 0.0025 | 0.341 | 0.627 | Other Abx (carbapenems) |
| Ghenea 2021^19^ | *Klebsiella spp* | Carbapenems (meropenem) | Carbapenems (meropenem) | TSA, regr | NR | 2 | NR | −0.0042 | NR | NR | −0.0077 to 0.0081 | 0.027 | 0.753 | Other Abx (carbapenems) |
| Gharbi 2015^39^ | *K. pneumoniae* | Carbapenems (meropenem) | OXA-48 (carbapenemase) production | TSA, CCA | UV | 1 yr | PCC | 0.71 | NR | NR | NR | 0.005 | NR | NR |
| Gharbi 2015^39^ | *K. pneumoniae* | Carbapenems (meropenem) | OXA-48 (carbapenemase) production | TSA, CCA | NR | 1 yr | NR | 1.07 | NR | NR | 0.10 to 2.05 | 0.03 | NR | NR |
| Ghenea 2021^19^ | *Klebsiella spp* | Carbapenems (imipenem) | Fluoroquinolones (ciprofloxacin) | TSA, regr | NR | 2 | NR | 0.001 | NR | NR | 0.0007 to 0.0014 | <0.001 | 0.753 | Other Abx |
| Ghenea 2021^19^ | *Klebsiella spp* | Carbapenems (imipenem) | Fluoroquinolones (ciprofloxacin) | TSA, regr | NR | 3 | NR | 0.0064 | NR | NR | 0.0004 to 0.0009 | <0.001 | 0.537 | Other Abx |
| Ghenea 2021^19^ | *Klebsiella spp* | Carbapenems (meropenem) | Fluoroquinolones (ciprofloxacin) | TSA, regr | NR | 2 | NR | −0.0002 | NR | NR | −0.0001 to 0.0010 | 0.094 | 0.753 | Other Abx |
| Ghenea 2021^19^ | *Klebsiella spp* | Carbapenems (meropenem) | Fluoroquinolones (ciprofloxacin) | TSA, regr | NR | 3 | NR | 0.0001 | NR | NR | −0.0002 to 0.0004 | 0.571 | 0.537 | Other Abx |
| Popovic 2020^31^ | *K. pneumoniae* | Carbapenems | Polymyxins (colistin) | CCA | UV | 0 yr | CCC | NR | NR | NR | NR | NR | 0.73 | NR |
| Popovic 2020^31^ | *K. pneumoniae* | Cephalosporins 1+2G | Carbapenems (ertapenem) | CCA | UV | 0 yr | CCC | NR | NR | NR | NR | NR | -0.71 | NR |
| Toth 2019^33^ | *K. pneumoniae;*  *K. oxytoca* | Cephalosporins | Cephalosporins | DR | NR | 4 | NR | 0.025 | NR | NR | 0.015 to 0.035 | <0.001 | NR | Prior resistance |
| Toth 2019^33^ | *K. pneumoniae;*  *K. oxytoca* | Cephalosporins | Cephalosporins | DR | NR | 5 | NR | 0.019 | NR | NR | 0.009 to 0.029 | <0.001 | NR | Prior resistance |
| Toth 2019^33^ | *K. pneumoniae;*  *K. oxytoca* | Cephalosporins | Cephalosporins | VAR | MV | 1-6 | HMSRRH | 0.196 | NR | NR | 0.027 to 0.319 | NR | NR | Prior resistance; other Abx |
| Popovic 2020^31^ | *K. pneumoniae* | Cephalosporins 1+2G | Polymyxins (colistin) | CCA | UV | 0 yr | CCC | NR | NR | NR | NR | NR | -0.84 | NR |
| Popovic 2020^31^ | *K. pneumoniae* | Cephalosporins 3+4G | Polymyxins (colistin) | CCA | UV | 0 yr | CCC | NR | NR | NR | NR | NR | -0.27 | NR |
| Popovic 2020^31^ | *K. pneumoniae* | Fluoroquinolones | Carbapenems (doripenem) | CCA | UV | 1 yr | CCC | NR | NR | NR | NR | NR | 0.74 | NR |
| Ghenea 2021^19^ | *Klebsiella spp* | Fluoroquinolones (ciprofloxacin) | Fluoroquinolones (ciprofloxacin) | TSA, regr | NR | 2 | NR | 0.0004 | NR | NR | 0.0001 to 0.0007 | 0.0047 | 0.753 | Other Abx |
| Ghenea 2021^19^ | *Klebsiella spp* | Fluoroquinolones (ciprofloxacin) | Fluoroquinolones (ciprofloxacin) | TSA, regr | NR | 3 | NR | −0.0001 | NR | NR | −0.0004 to 0.0001 | 0.33 | 0.537 | Other Abx |
| Popovic 2020^31^ | *K. pneumoniae* | Fluoroquinolones | Polymyxins (colistin) | CCA | UV | 1 yr | CCC | NR | NR | NR | NR | NR | -0.66 | NR |
| Popovic 2020^31^ | *K. pneumoniae* | Penicillin/B-lact inhib | Polymyxins (colistin) | CCA | UV | 1 yr | CCC | NR | NR | NR | NR | NR | -0.76 | NR |
| Popovic 2020^31^ | *K. pneumoniae* | Polymyxin (colistin) | Carbapenems (doripenem) | CCA | UV | 1 yr | CCC | NR | NR | NR | NR | NR | 0.48 | NR |
| Popovic 2020^31^ | *K. pneumoniae* | Polymyxin (colistin) | Carbapenems (ertapenem) | CCA | UV | 0 yr | CCC | NR | NR | NR | NR | NR | 0.6 | NR |
| Popovic 2020^31^ | *K. pneumoniae* | Polymyxin (colistin) | Carbapenems (meropenem) | CCA | UV | 1 yr | CCC | NR | NR | NR | NR | NR | 0.48 | NR |
| Popovic 2020^31^ | *K. pneumoniae* | Polymyxin (colistin) | Polymyxin (colistin) | CCA | UV | 0 yr | CCC | NR | NR | NR | NR | NR | 0.89 | NR |
| ^a^Lag in months unless otherwise stated.  **Abbreviations:** 1/2/3/4G, 1^st^/2^nd^/3^rd^/4^th^ generation; Abx, antibiotics; ARIMA, autoregressive integrated moving average; B-lact inhib, B-lactamase inhibitor; BV, bivariate; CCA, Cross correlation analysis; CCC, cross-correlation coefficient; CI, confidence interval; coeff, coefficient; comm, community; DR, dynamic regression; HMSRRH, highest magnitude of significant response in the response horizon; hosp, hospital; *K. pneumoniae*, *Klebsiella pneumoniae*; LTF, linear transfer function; mo, months; MV, multivariate; MVA, multivariate analysis; MVR, multivariate regression; PCC, Pearson correlation coefficient; prev, prevalence; qtr, quarters; RC, regression coefficient; regr, regression; SE, standard error; spp, species; TSA, time series analysis; UV, univariate; VAR, vector autoregressive model; yr, years. | | | | | | | | | | | | | | |

**Time-lag results: *S. aureus* (ecological study design)**

| **Study** | **Pathogen** | **Antibiotic use** | **Resistance to/ Resistance mechanism** | **Statistics** | **Model** | **Lag (mo)^a^** | **Coefficient** | | | | | | **R^2^** | **Adjusted variables** |
| --- | --- | --- | --- | --- | --- | --- | --- | --- | --- | --- | --- | --- | --- | --- |
|  |  |  |  |  |  |  | **Type** | **Coeff** | **SE** | **T-ratio** | **95% CI** | **p-value** |  |  |
| ***Staphylococcus aureus* (MRSA)** | | | | | | | | | | | | | | |
| Berger 2004^38^ | *S. aureus* | Cephalosporins 3G [all units] | Fluoroquinolones | GAM | NR | 0 | NR | NR | NR | NR | NR | NR | NR | NR |
| Kaier 2009b^22^ | MRSA | Cephalosporins 2G | MRSA incidence (B-lactam Abx: methicillin, oxacillin, cephalosporin) | TSA, regr | MV | 1 | NR | 1.41 | NR | 2.39 | NR | 0.023 | 0.66 | Prior resistance; other Abx; MRSA admissions; alcohol hand rub |
| Kaier 2009b^22^ | MRSA | Cephalosporins 3G | MRSA incidence (B-lactam Abx: methicillin, oxacillin, cephalosporin) | TSA, regr | MV | 3-4 | NR | 1.03 | NR | 2.03 | NR | 0.05 | 0.66 | Prior resistance; other Abx; MRSA admissions; alcohol hand rub |
| Aldeyab 2008^13^ | MRSA | Cephalosporins 3G | MRSA incidence | TSA, LTR, DR | MV | 2 | NR | 0.0273 | 0.00449 | NR | NR | <0.0001 | 0.784 | Other Abx; infection control; alcohol hand rub |
| Lopez-Lozano 2019^26^ | MRSA | Cephalosporins 3G | MRSA incidence | TSA, MVR | MV | 3 | NR | 0.026 | NR | 4.51 | 0.015 to 0.037 | 0.0005 | 0.561 | Prior resistance; other Abx |
| Monnet 2004^30^ | MRSA | Cephalosporins 3G | MRSA incidence | TSA, MVA, PDL | MV | 4-7 | NR | 0.29 | NR | 2.75 | NR | 0.009 | 0.902 | Prior resistance; other Abx |
| Vernaz 2008^35^ | MRSA | Cephalosporins 3G | MRSA incidence | TSA, ARIMA, LTF | MV | 4 | NR | 0.014 | 0.006 | 2.15 | NR | 0.035 | 0.57 | Prior resistance; other Abx (hosp+comm) |
| Vernaz 2008^35^ | MRSA | Cephalosporins 3G | MRSA incidence | TSA, ARIMA, LTF | MV | 5 | NR | 0.015 | 0.007 | 2.21 | NR | 0.031 | 0.57 | Prior resistance; other Abx (hosp+comm) |
| Lawes 2015^24^ | MRSA | Cephalosporins 3G | CC22 MRSA strain | TSA, MARS | MV | 5 | Chg PD | -0.007 | NR | -4.22 | -0.010 to -0.004 | <0.001 | 0.912 | Other Abx; CC30 prev; bed occupancy; LoS; hand-hygiene; MRSA screening; MRSA admissions |
| Lawes 2015^24^ | MRSA | Cephalosporins 3G | CC30 MRSA strain | TSA, MARS | MV | 5 | Chg PD | -0.008 | NR | -3.74 | -0.013 to -0.003 | 0.001 | 0.94 | Other Abx; CC22 prev; bed occupancy; LoS; MRSA screening; MRSA admissions |
| Lawes 2015^24^ | MRSA | Cephalosporins 3G | CC5/Other MRSA Strain | TSA, MARS | MV | 5 | Chg PD | -0.004 | NR | -3.69 | -0.006 to -0.002 | <0.001 | 0.583 | Other Abx; LoS; MRSA screening; MRSA admissions |
| Lawes 2015^24^ | MRSA | Fluoroquinolones | Fluoroquinolones (ciprofloxacin) | TSA | UV | 0 | NR | 0.062 | NR | 3.36 | 0.027 to 0.097 | 0.001 | 0.451 | NR |
| Lawes 2015^24^ | MRSA | Fluoroquinolones | Fluoroquinolones (ciprofloxacin) | TSA | UV | 0 | NR | 0.128 | NR | 3.14 | 0.048 to 0.209 | 0.002 | 0.331 | NR |
| Lawes 2015^24^ | MRSA | Fluoroquinolones | Fluoroquinolones (ciprofloxacin) | TSA | UV | 0 | NR | 0.108 | NR | 6.58 | 0.076 to 0.140 | <0.001 | 0.074 | NR |
| Berger 2004^38^ | *S. aureus* | Fluoroquinolones [all units] | Fluoroquinolones | GAM | NR | 4 | NR | NR | NR | NR | NR | NR | NR | NR |
| Berger 2004^38^ | *S. aureus* | Fluoroquinolones [medicine unit] | Fluoroquinolones | GAM | NR | 0-4 | NR | NR | NR | NR | NR | NR | NR | NR |
| Berger 2004^38^ | *S. aureus* | Fluoroquinolones [surgery unit] | Fluoroquinolones | GAM | NR | 2 | NR | NR | NR | NR | NR | NR | NR | NR |
| Aldeyab 2008^13^ | MRSA | Fluoroquinolones | MRSA incidence | TSA, DR, LTF | MV | 1 | NR | 0.00481 | 0.00098 | NR | NR | <0.0001 | 0.784 | Other Abx; infection control, alcohol hand rub |
| Lopez-Lozano 2019^26^ | MRSA | Fluoroquinolones | MRSA incidence | TSA, MVA | MV | 3 | NR | 0.003 | NR | 2.13 | 0.000 to 0.006 | 0.0299 | 0.561 | Prior resistance; other Abx |
| Mahamat 2007^29^ | MRSA | Fluoroquinolones | MRSA incidence | TSA, DR | MV | 2 | NR | 3.2 | NR | NR | NR | <0.001 | NR | Alcohol-based hand rub |
| Monnet 2004^30^ | MRSA | Fluoroquinolones | MRSA incidence | TSA, MVA, PDL | MV | 4-5 | NR | 0.255 | NR | 3.43 | NR | 0.002 | 0.902 | Prior resistance; other Abx |
| Vernaz 2008^35^ | MRSA | Fluoroquinolones | MRSA incidence | TSA, ARIMA, LTF | MV | 1 | NR | 0.01 | 0.004 | 2.71 | NR | 0.009 | 0.57 | Prior resistance; other Abx (hosp+comm) |
| Kaier 2009b^22^ | MRSA | Fluoroquinolones | MRSA incidence (B-lactam Abx: methicillin, oxacillin, cephalosporin) | TSA, regr | MV | 4 | NR | 1.12 | NR | 2.73 | NR | 0.01 | 0.66 | Prior resistance; other Abx; MRSA admissions; alcohol hand rub |
| Lawes 2015^24^ | MRSA | Fluoroquinolones, 1st order term | CC22 MRSA strain | TSA, MARS | MV | 2 | Chg PD | 0.033 | NR | 2.69 | 0.009 to 0.057 | 0.016 | 0.912 | Other Abx; CC30 prev; bed occupancy; LoS; hand-hygiene; MRSA screening; MRSA admissions |
| Lawes 2015^24^ | MRSA | Fluoroquinolones, 2nd order term | CC22 MRSA strain | TSA, MARS | MV | 2 | Chg PD | -0.032 | NR | -2.62 | -0.055 to 0.009 | 0.019 | 0.912 | Other Abx; CC30 prev; bed occupancy; LoS; hand-hygiene; MRSA screening; MRSA admissions |
| Lawes 2015^24^ | MRSA | Fluoroquinolones, 1st order term | CC30 MRSA strain | TSA, MARS | MV | 4 | Chg PD | -0.049 | NR | -4.38 | -0.071 to -0.027 | <0.001 | 0.94 | Other Abx; CC22 prev; bed occupancy; LoS; MRSA screening; MRSA admissions |
| Lawes 2015^24^ | MRSA | Fluoroquinolones, 2nd order term | CC30 MRSA strain | TSA, MARS | MV | 4 | Chg PD | 0.018 | NR | 3.92 | 0.017 to 0.019 | <0.001 | 0.94 | Other Abx; CC22 prev; bed occupancy; LoS; MRSA screening; MRSA admissions |
| Lawes 2015^24^ | MRSA | Fluoroquinolones, 3rd order term | CC30 MRSA strain | TSA, MARS | MV | 4 | Chg PD | -0.021 | NR | -4.16 | -0.031 to -0.011 | <0.001 | 0.94 | Other Abx; CC22 prev; bed occupancy; LoS; MRSA screening; MRSA admissions |
| Lawes 2015^24^ | MRSA | Lincosamides (clindamycin) | Lincosamides (clindamycin) | TSA | UV | 0 | NR | 0.173 | NR | 9.76 | 0.137 to 0.208 | <0.001 | 0.298 | NR |
| Lawes 2015^24^ | MRSA | Lincosamides (clindamycin) | Lincosamides (clindamycin) | TSA | UV | 0 | NR | 0.455 | NR | 2.3 | 0.067 to 0.843 | 0.023 | 0.691 | NR |
| Lawes 2015^24^ | MRSA | Lincosamides (clindamycin) | Lincosamides (clindamycin) | TSA | UV | 0 | NR | 0.334 | NR | 4.11 | 0.175 to 0.493 | <0.001 | 0.176 | NR |
| Kaier 2009b^22^ | MRSA | Lincosamides | MRSA incidence (B-lactam Abx: methicillin, oxacillin, cephalosporin) | TSA, regr | MV | 2 | NR | 0.42 | NR | 2.04 | NR | 0.05 | 0.66 | Prior resistance; other Abx; MRSA admissions; alcohol hand rub |
| Berger 2004^38^ | *S. aureus* | Penicillins (oxacillin) [all units] | Fluoroquinolones | GAM | NR | 3 | NR | NR | NR | NR | NR | NR | NR | NR |
| Berger 2004^38^ | *S. aureus* | Penicillins (oxacillin) [ICU] | Fluoroquinolones | GAM | NR | 3 | NR | NR | NR | NR | NR | NR | NR | NR |
| Berger 2004^38^ | *S. aureus* | Penicillin/B-lact inhib [medicine unit] | Fluoroquinolones | GAM | NR | 0-5 | NR | NR | NR | NR | NR | NR | NR | NR |
| Lopez-Lozano 2019^26^ | MRSA | Penicillin/B-lact inhib (co-amoxiclav) | MRSA incidence | TSA, MVA | MV | 1 | NR | 0.003 | NR | 4.39 | 0.002 to 0.005 | <0.0001 | 0.561 | Prior resistance; other Abx |
| Vernaz 2008^35^ | MRSA | Penicillin/B-lact inhib (piperacillin/tazobactam) | MRSA incidence | TSA, ARIMA, LTF | MV | 3 | NR | 0.041 | 0.014 | 2.97 | NR | 0.004 | 0.57 | Prior resistance; other Abx (hosp+comm) |
| Aldeyab 2008^13^ | MRSA | Penicillin/B-lact inhib [amoxicillin/clavulanic acid] | MRSA incidence | TSA, LTF, DR | MV | 1 | NR | 0.00349 | 0.000651 | NR | NR | <0.0001 | 0.784 | Other Abx; infection control; alcohol hand rub |
| Lawes 2015^24^ | MRSA | Penicillin/B-lact inhib (co-amoxiclav) | CC22 MRSA strain | TSA, MARS | MV | 2 | Chg PD | 0.01 | NR | 3.1 | 0.004 to 0.016 | 0.007 | 0.912 | Other Abx; CC30 prev; bed occupancy; LoS; hand-hygiene; MRSA screening; MRSA admissions |
| Lawes 2015^24^ | MRSA | Penicillin/B-lact inhib (co-amoxiclav), 1st order term | CC30 MRSA strain | TSA, MARS | MV | 5 | Chg PD | -0.005 | NR | -3.35 | -0.008 to -0.002 | 0.003 | 0.94 | Other Abx; CC22 prev; bed occupancy; LoS; MRSA screening; MRSA admissions |
| Lawes 2015^24^ | MRSA | Penicillin/B-lact inhib (co-amoxiclav), 2nd order term | CC30 MRSA strain | TSA, MARS | MV | 5 | Chg PD | -0.003 | NR | -3.82 | -0.005 to -0.001 | <0.001 | 0.94 | Other Abx; CC22 prev; bed occupancy; LoS; MRSA screening; MRSA admissions |
| Lawes 2015^24^ | MRSA | Penicillin/B-lact inhib (co-amoxiclav) | CC5/Other MRSA Strain | TSA, MARS | MV | 5 | Chg PD | 0.008 | NR | 6.07 | 0.005 to 0.013 | <0.001 | 0.583 | Other Abx; LoS; MRSA screening; MRSA admissions |
| ^a^Lag in months unless otherwise stated.  **Abbreviations:** 1/2/3/4G, 1^st^/2^nd^/3^rd^/4^th^ generation; Abx, antibiotics; ARIMA, autoregressive integrated moving average; B-lact inhib, B-lactamase inhibitor; BV, bivariate; CCA, Cross correlation analysis; ceph, cephalosporins; chg, change; CI, confidence interval; clav, clavulanic acid; coeff, coefficient; comm, community; DR, dynamic regression; GAM, generalised additive model; hosp, hospital; ICU, intensive care unit; LoS, length of stay; LTF, linear transfer function; MARS, Multivariate Adaptive Regression Spline model; mo, months; MRSA, methicillin-resistant *Staphylococcus aureus*; MV, multivariate; MVA, multivariate analysis; MVR, multivariate regression; prevD, prevalence density; PDL, polynomial distributed lag modelling; prev, prevalence; qtr, quarters; RC, regression coefficient; regr, regression; *S. aureus*, *Staphylococcus aureus*; SE, standard error; spp, species; TSA, time series analysis; UV, univariate; VAR, vector autoregressive model; yr, years. | | | | | | | | | | | | | | |

**Time-lag results: Enterococci (ecological study design)**

| **Study** | **Pathogen** | **Antibiotic use** | **Resistance to/ Resistance mechanism** | **Statistics** | **Model** | **Lag (mo)^a^** | **Coefficient** | | | | | | **R^2^** | **Adjusted variables** |
| --- | --- | --- | --- | --- | --- | --- | --- | --- | --- | --- | --- | --- | --- | --- |
|  |  |  |  |  |  |  | **Type** | **Coeff** | **SE** | **T-ratio** | **95% CI** | **p-value** |  |  |
| ***Enterococcus* (VRE)** | | | | | | | | | | | | | | |
| Remschmidt 2017^32^ | Enterococcus (VRE) (*E. faecium + E. faecalis*) [colonised/ infected] | Glycopetides | VRE incidence | GEE | MV | 1 | Adj IRR | 1.02 | NA | NA | 1.01–1.04 | 0.002 | NA | Other Abx, ward type, VRE on admission, screening, year |
| Remschmidt 2017^32^ | Enterococcus (VRE) (*E. faecium + E. faecalis*) [infected only] | Glycopetides | VRE incidence | GEE | MV | 1 | Adj IRR | 1.03 | NA | NA | 1.01–1.05 | 0.0038 | NA | Ward type, VRE on admission, year |
| Kritsotakis 2008^23^ | Enterococcus (VRE) (except *E. gallinarum* or *E. casseliflavus*) | Glycopeptides | VRE incidence | TSA, LTF | MV | 2 | RC | 0.024 | 0.009 | 2.8 | NR | 0.008 | NR | Prior resistance; other Abx |
| Kritsotakis 2008^23^ | Enterococcus (VRE) (except *E. gallinarum* or *E. casseliflavus*) | Penicillin/B-lact inhib | VRE incidence | TSA, LTF | MV | 6 | RC | -0.01 | 0.005 | -2.1 | NR | 0.043 | NR | Prior resistance; other Abx |
| ^a^Lag in months unless otherwise stated.  **Abbreviations:** Abx, antibiotics; adj, adjusted; ARIMA, autoregressive integrated moving average; B-lact inhib, B-lactamase inhibitor; CCA, Cross correlation analysis; CI, confidence interval; coeff, coefficient; comm, community; DR, dynamic regression; GEE, generalised estimating equation model; hosp, hospital; IRR, incidence rate ratio; LTF, linear transfer function; mo, months; MV, multivariate; MVA, multivariate analysis; MVR, multivariate regression; prev, prevalence; qtr, quarters; RC, regression coefficient; regr, regression; SE, standard error; spp, species; TSA, time series analysis; UV, univariate; VAR, vector autoregressive model; VRE, vancomycin-resistant *Enterococcus*; yr, years. | | | | | | | | | | | | | | |

**Time-lag results: *P*. *aeruginosa* (ecological study design)**

| **Study** | **Pathogen** | **Antibiotic use** | **Resistance to/ Resistance mechanism** | **Statistics** | **Model** | **Lag (mo)^a^** | **Coefficient** | | | | | | **R^2^** | **Adjusted variables** |
| --- | --- | --- | --- | --- | --- | --- | --- | --- | --- | --- | --- | --- | --- | --- |
|  |  |  |  |  |  |  | **Type** | **Coeff** | **SE** | **T-ratio** | **95% CI** | **p-value** |  |  |
| ***Pseudomonas aeruginosa*** | | | | | | | | | | | | | | |
| Lopez-Lozano 2019^26^ | *P. aeruginosa* | Aminoglycosides (gentamicin, tobramycin) | Aminoglycosides (gentamicin) | TSA, MVA | MV | 1 | NR | 0.221 | NR | 4.18 | 0.077 to 0.365 | <0.0001 | 0.857 | Prior resistance; other Abx |
| Hocquet 2008^20^ | *P. aeruginosa* | Aminoglycosides (amikacin, tobramycin, gentamicin) | Overproduction of MexXY-OprM^b^ | TSA, ARIMA, LTF | MV | 0, 3, 4, 6 | NR | 0.142 | 0.038 | NR | NR | NR | 0.811 | Prior resistance; other Abx |
| Willmann 2013^37^ | *P. aeruginosa* | Carbapenems (meropenem) | Aminoglycosides (tobramycin) | TSA, LTF | MV | 0 qtr | NR | 0.16 | NR | NR | 0.05 to 0.28 | 0.006 | NR | Other Abx |
| Baditoiu 2017^15^ | *P. aeruginosa* | Carbapenems | Carbapenems | CCA, regr | NR | 0 qtr | NR | 0.046 | 0.008 | NR | NR | <0.001 | 0.861 | None |
| Toth 2019^33^ | *P. aeruginosa* | Carbapenems | Carbapenems | DR | NR | 0 | NR | 0.087 | NR | NR | 0.060 to 0.114 | <0.001 | NR | Prior resistance |
| Toth 2019^33^ | *P. aeruginosa* | Carbapenems | Carbapenems | DR | NR | 1 | NR | 0.109 | NR | NR | 0.082 to 0.136 | <0.001 | NR | Prior resistance |
| Toth 2019^33^ | *P. aeruginosa* | Carbapenems | Carbapenems | VAR | BV | 2 | HMSRRH | 0.127 | NR | NR | 0.012 to 0.234 | NR | NR | Prior resistance |
| Toth 2019^33^ | *P. aeruginosa* | Carbapenems | Carbapenems | VAR | MV | 1 to 2 | HMSRRH | 0.072 | NR | NR | 0.012 to 0.176 | NR | NR | Prior resistance; other Abx |
| Beovic 2011^16^ | *P. aeruginosa* | Carbapenems [AS unit] | Carbapenems | TSA, ARIMA, CCA | NR | 1 | NR | NR | NR | NR | NR | NR | NR | NR |
| Beovic 2011^16^ | *P. aeruginosa* | Carbapenems [ID unit] | Carbapenems | TSA, ARIMA, CCA | NR | 6 | NR | NR | NR | NR | NR | NR | NR | NR |
| Beovic 2011^16^ | *P. aeruginosa* | Carbapenems (ertapenem) [AS unit] | Carbapenems | TSA, ARIMA, CCA | NR | 1 | NR | NR | NR | NR | NR | NR | NR | NR |
| Beovic 2011^16^ | *P. aeruginosa* | Carbapenems (ertapenem) [SICU] | Carbapenems | TSA, ARIMA, CCA | NR | 2 | NR | NR | NR | NR | NR | NR | NR | NR |
| Beovic 2011^16^ | *P. aeruginosa* | Carbapenems (imipenem) [ID unit] | Carbapenems | TSA, ARIMA, CCA | NR | 6 | NR | NR | NR | NR | NR | NR | NR | NR |
| Lepper 2002^25^ | *P. aeruginosa* | Carbapenems (imipenem) | Carbapenems (imipenem) | PCC | MV | 0 | PCC | 0.58 | NR | NR | NR | ≤0.005 | NR | Other Abx; seasonal effects; patient transfers |
| Lepper 2002^25^ | *P. aeruginosa* | Carbapenems (imipenem) | Carbapenems (imipenem) | PCC | MV | 1 | PCC | 0.63 | NR | NR | NR | ≤0.005 | NR | Other Abx; seasonal effects; patient transfers |
| Lepper 2002^25^ | *P. aeruginosa* | Carbapenems (imipenem) | Carbapenems (imipenem) | TSA | MV | 0 | RC | NR | NR | NR | NR | <0.1 | NR | Other Abx; seasonal effects; patient transfers |
| Lepper 2002^25^ | *P. aeruginosa* | Carbapenems (imipenem) | Carbapenems (imipenem) | TSA | MV | 1 | RC | NR | NR | NR | NR | <0.05 | NR | Other Abx; seasonal effects; patient transfers |
| Lopez-Lozano 2000^27^ | *P. aeruginosa* | Carbapenems (imipenem) | Carbapenems (imipenem) | TSA, LTF | NR | 1 | NR | 0.4 | 0.104 | 3.83 | NR | <0.001 | 0.63 | Prior resistance |
| Baditoiu 2017^15^ | *P. aeruginosa* | Carbapenems (meropenem) | Carbapenems | CCA, regr | NR | 0 qtr | NR | 0.041 | 0.011 | NR | NR | 0.038 | 0.921 | Current+prior resistance |
| Baditoiu 2017^15^ | *P. aeruginosa* | Carbapenems (meropenem) | Carbapenems | CCA, regr | NR | 1 qtr | NR | 0.047 | 0.018 | NR | NR | 0.078 | 0.921 | Current+prior resistance |
| Beovic 2011^16^ | *P. aeruginosa* | Carbapenems (meropenem) [ID unit] | Carbapenems | TSA, ARIMA, CCA | NR | 6 | NR | NR | NR | NR | NR | NR | NR | NR |
| Erdeljic 2011^17^ | *P. aeruginosa* | Carbapenems (meropenem) | Carbapenems (imipenem) | TSA | UV | 0 | RC | 0.879 | 0.275 | 3.922 | NR | 0.002 | NR | NR |
| Erdeljic 2011^17^ | *P. aeruginosa* | Carbapenems (meropenem) | Carbapenems (meropenem) | TSA | UV | 0 | RC | 0.876 | 0.277 | 3.937 | NR | 0.002 | NR | NR |
| Erdeljic 2011^17^ | *P. aeruginosa* | Carbapenems (meropenem) | Carbapenems (meropenem) (MDR) | TSA | UV | 0 | RC | 0.992 | 0.221 | 4.492 | NR | 0.0006 | NR | NR |
| Willmann 2013^37^ | *P. aeruginosa* | Carbapenems (meropenem) | Carbapenems (meropenem) | TSA, LTF | UV | 1 qtr | NR | 0.24 | NR | NR | 0.12 to 0.35 | <0.001 | NR | NA |
| Aldrin 2013^14^ | *P. aeruginosa* | Carbapenems (meropenem + imipenem) | Carbapenems (meropenem) | Binomial regr | NR | 0 | RC | 0.0184 | NR | NR | NR | <0.05 | NR | Prior resistance |
| Aldrin 2013^14^ | *P. aeruginosa* | Carbapenems (meropenem + imipenem) | Carbapenems (meropenem) | Binomial regr | NR | 1 | RC | 0.0184 | NR | NR | NR | <0.05 | NR | Prior resistance |
| Lepper 2002^25^ | *P. aeruginosa* | Carbapenems (imipenem) | Cephalosporins 3G (ceftazidime) | PCC | MV | 0 | PCC | 0.52 | NR | NR | NR | ≤0.005 | NR | Other Abx; seasonal effects; patient transfers |
| Lepper 2002^25^ | *P. aeruginosa* | Carbapenems (imipenem) | Cephalosporins 3G (ceftazidime) | PCC | MV | 1 | PCC | 0.56 | NR | NR | NR | ≤0.005 | NR | Other Abx; seasonal effects; patient transfers |
| Lepper 2002^25^ | *P. aeruginosa* | Carbapenems (imipenem) | Cephalosporins 3G (ceftazidime) | TSA | MV | 0 | RC | NR | NR | NR | NR | <0.06 | NR | Other Abx; seasonal effects; patient transfers |
| Willmann 2013^37^ | *P. aeruginosa* | Carbapenems (meropenem) | Fluoroquinolones (ciprofloxacin) | TSA, LTF | MV | 0 qtr | NR | 0.15 | NR | NR | -0.06 to 0.36 | 0.15 | NR | Other Abx |
| Willmann 2013^37^ | *P. aeruginosa* | Carbapenems (meropenem) | Fluoroquinolones (ciprofloxacin) | TSA, LTF | MV | 0 qtr | NR | 0.2 | NR | NR | -0.03 to 0.42 | 0.088 | NR | Other Abx + cefepime |
| Lepper 2002^25^ | *P. aeruginosa* | Carbapenems (imipenem) | Penicillin/B-lact inhib (piperacillin/ tazobactam) | PCC | MV | 0 | PCC | 0.57 | NR | NR | NR | ≤0.005 | NR | Other Abx; seasonal effects; patient transfers |
| Lepper 2002^25^ | *P. aeruginosa* | Carbapenems (imipenem) | Penicillin/B-lact inhib (piperacillin/ tazobactam) | PCC | MV | 1 | PCC | 0.57 | NR | NR | NR | ≤0.005 | NR | Other Abx; seasonal effects; patient transfers |
| Lepper 2002^25^ | *P. aeruginosa* | Carbapenems (imipenem) | Penicillin/B-lact inhib (piperacillin/ tazobactam) | TSA | MV | 0 | RC | NR | NR | NR | NR | <0.01 | NR | Other Abx; seasonal effects; patient transfers |
| Hocquet 2008^20^ | *P. aeruginosa* | Carbapenems (imipenem, ertapenem, meropenem) | Overproduction of MexXY-OprM^b^ | TSA, ARIMA, LTF | MV | 0, 2, 5, 6 | NR | -0.158 | 0.046 | NR | NR | NR | 0.811 | Prior resistance; other Abx |
| Willmann 2013^37^ | *P. aeruginosa* | Carbapenems (meropenem) | 3/4 MDR^c^ *P*. *aeruginosa* | TSA, LTF (all Abx) | UV | 1 qtr | NR | 0.28 | NR | NR | NR | <0.001 | NR | NR |
| Willmann 2013^37^ | *P. aeruginosa* | Carbapenems (meropenem) | 3/4 MDR^c^ *P*. *aeruginosa* | TSA, LTF (all Abx) | MV | 1 qtr | NR | 0.15 | NR | NR | 0.015 to 0.29 | 0.03 | NR | Other Abx |
| Willmann 2013^37^ | *P. aeruginosa* | Carbapenems (meropenem) | 3/4 MDR^c^ *P*. *aeruginosa* | TSA, LTF (all Abx) | MV | 1 qtr | NR | 0.13 | NR | NR | -0.02 to 0.28 | 0.086 | NR | Other Abx (cefepime) |
| Willmann 2013^37^ | *P. aeruginosa* | Cephalosporins 3G | Aminoglycosides (tobramycin) | TSA, LTF | MV | 0 qtr | NR | 0.12 | NR | NR | 0.01 to 0.23 | 0.029 | NR | Other Abx |
| Lepper 2002^25^ | *P. aeruginosa* | Cephalosporins 3G (ceftazidime) | Carbapenems (imipenem) | PCC | MV | 0 | PCC | -0.24 | NR | NR | NR | NR | NR | Other Abx; seasonal effects; patient transfers |
| Lepper 2002^25^ | *P. aeruginosa* | Cephalosporins 3G (ceftazidime) | Carbapenems (imipenem) | PCC | MV | 1 | PCC | -0.07 | NR | NR | NR | NR | NR | Other Abx; seasonal effects; patient transfers |
| Willmann 2013^37^ | *P. aeruginosa* | Cephalosporins | Cephalosporins 3G (ceftazidime) | TSA, LTF | MV | 0 qtr | NR | 0.04 | NR | NR | 0.0006 to 0.09 | 0.047 | NR | Other Abx + cefepime |
| Lepper 2002^25^ | *P. aeruginosa* | Cephalosporins 3G (ceftazidime) | Cephalosporins 3G (ceftazidime) | PCC | MV | 0 | PCC | 0.08 | NR | NR | NR | NR | NR | Other Abx; seasonal effects; patient transfers |
| Lepper 2002^25^ | *P. aeruginosa* | Cephalosporins 3G (ceftazidime) | Cephalosporins 3G (ceftazidime) | PCC | MV | 1 | PCC | -0.26 | NR | NR | NR | NR | NR | Other Abx; seasonal effects; patient transfers |
| Willmann 2013^37^ | *P. aeruginosa* | Cephalosporins 3G (ceftriaxone) | Cephalosporins 3G (ceftazidime) | TSA, LTF | MV | 2 qtr | NR | 0.24 | NR | NR | -0.22 to 0.71 | 0.3 | NR | Other Abx |
| Willmann 2013^37^ | *P. aeruginosa* | Cephalosporins 4G (cefepime) | Cephalosporins 3G (ceftazidime) | TSA, LTF | MV | 0 qtr | NR | 0.41 | NR | NR | 0.15 to 0.66 | 0.002 | NR | Other Abx |
| Erdeljic 2011^17^ | *P. aeruginosa* | Cephalosporins 4G (cefepime) | Cephalosporins 4G (cefepime) | TSA | UV | 2 | RC | 0.962 | 0.041 | 5.035 | NR | 0.001 | NR | NR |
| Willmann 2013^37^ | *P. aeruginosa* | Cephalosporins | Fluoroquinolones (ciprofloxacin) | TSA, LTF | MV | 0 qtr | NR | 0.05 | NR | NR | -0.004 to 0.1 | 0.069 | NR | Other Abx + cefepime |
| Willmann 2013^37^ | *P. aeruginosa* | Cephalosporins 3G | Fluoroquinolones (ciprofloxacin) | TSA, LTF | MV | 0 qtr | NR | 0.18 | NR | NR | -0.1 to 0.46 | 0.2 | NR | Other Abx |
| Willmann 2013^37^ | *P. aeruginosa* | Cephalosporins 4G (cefepime) | Fluoroquinolones (ciprofloxacin) | TSA, LTF | MV | 0 qtr | NR | 0.45 | NR | NR | -0.17 to 1.06 | 0.16 | NR | Other Abx |
| Lepper 2002^25^ | *P. aeruginosa* | Cephalosporins 3G (ceftazidime) | Penicillin/B-lact inhib (piperacillin/ tazobactam) | PCC | MV | 0 | PCC | -0.13 | NR | NR | NR | NR | NR | Other Abx; seasonal effects; patient transfers |
| Lepper 2002^25^ | *P. aeruginosa* | Cephalosporins 3G (ceftazidime) | Penicillin/B-lact inhib (piperacillin/ tazobactam) | PCC | MV | 1 | PCC | -0.25 | NR | NR | NR | NR | NR | Other Abx; seasonal effects; patient transfers |
| Hocquet 2008^20^ | *P. aeruginosa* | Cephalosporins, antipseudomonal (3+4G) (ceftazidime, cefepime) | Overproduction of MexXY-OprM^b^ | TSA, ARIMA, LTF | MV | 2 | NR | 0.037 | 0.007 | NR | NR | NR | 0.811 | Prior resistance; other Abx |
| Hocquet 2008^20^ | *P. aeruginosa* | Cephalosporins, inactive on P. aeruginosa (2+3G) (cefuroxime, cefoxitin, ceftriaxone, cefotaxime) | Overproduction of MexXY-OprM^b^ | TSA, ARIMA, LTF | MV | 0, 2, 6 | NR | -0.083 | 0.014 | NR | NR | NR | 0.811 | Prior resistance; other Abx |
| Lepper 2002^25^ | *P. aeruginosa* | Cephalosporins 3G (ceftazidime) | Any of imipenem, ceftazidime or piperacillin-tazobactam | TSA | MV | ≥6 | RC | NR | NR | NR | NR | NR | NR | Other Abx; seasonal effects; patient transfers |
| Willmann 2013^37^ | *P. aeruginosa* | Cephalosporins | 3/4 MDR^c^ *P*. *aeruginosa* | TSA, LTF | UV | 0 qtr | NR | 0.05 | NR | NR | NR | <0.001 | NR | NR |
| Willmann 2013^37^ | *P. aeruginosa* | Cephalosporins (expanded-spectrum) | 3/4 MDR^c^ *P*. *aeruginosa* | TSA, LTF | UV | 1 qtr | NR | 0.35 | NR | NR | NR | <0.001 | NR | NR |
| Willmann 2013^37^ | *P. aeruginosa* | Cephalosporins (expanded-spectrum) | 3/4 MDR^c^ *P*. *aeruginosa* | TSA, LTF | MV | 1 qtr | NR | 0.18 | NR | NR | -0.09 to 0.45 | 0.2 | NR | Other Abx |
| Willmann 2013^37^ | *P. aeruginosa* | Cephalosporins (expanded-spectrum) | 3/4 MDR^c^ *P*. *aeruginosa* | TSA, LTF | MV | 1 qtr | NR | 0.11 | NR | NR | -0.16 to 0.36 | 0.42 | NR | Other Abx (cefepime) |
| Willmann 2013^37^ | *P. aeruginosa* | Cephalosporins 3G (ceftriaxone) | 3/4 MDR^c^ *P*. *aeruginosa* | TSA, LTF | UV | 1 qtr | NR | 0.6 | NR | NR | NR | 0.001 | NR | NR |
| Willmann 2013^37^ | *P. aeruginosa* | Cephalosporins 4G (cefepime) | 3/4 MDR^c^ *P*. *aeruginosa* | TSA, LTF | UV | 0 qtr | NR | 0.6 | NR | NR | NR | <0.001 | NR | NR |
| Willmann 2013^37^ | *P. aeruginosa* | Cephalosporins 4G (cefepime) | 3/4 MDR^c^ *P*. *aeruginosa* | TSA, LTF | MV | 0 qtr | NR | 0.22 | NR | NR | -0.02 to 0.65 | 0.3 | NR | Other Abx (cefepime) |
| Willmann 2013^37^ | *P. aeruginosa* | Cephalosporins | XDR *P aeruginosa* | TSA, LTF | MV | 0 qtr | NR | 0.03 | NR | NR | 0.02 to 0.05 | <0.001 | NR | Other Abx (cefepime) |
| Willmann 2013^37^ | *P. aeruginosa* | Cephalosporins | XDR *P aeruginosa* | TSA, LTF | UV | 0 qtr | NR | 0.04 | NR | NR | NR | <0.001 | NR | NR |
| Willmann 2013^37^ | *P. aeruginosa* | Cephalosporins (expanded-spectrum) | XDR *P aeruginosa* | TSA, LTF | MV | 0 qtr | NR | 0.15 | NR | NR | 0.02 to 0.28 | 0.025 | NR | Other Abx |
| Willmann 2013^37^ | *P. aeruginosa* | Cephalosporins (expanded-spectrum) | XDR *P aeruginosa* | TSA, LTF | UV | 0 qtr | NR | 0.27 | NR | NR | NR | <0.001 | NR | NR |
| Willmann 2013^37^ | *P. aeruginosa* | Cephalosporins 4G (cefepime) | XDR *P aeruginosa* | TSA, LTF | MV | 0 qtr | NR | 0.25 | NR | NR | -0.02 to 0.52 | 0.067 | NR | Other Abx |
| Willmann 2013^37^ | *P. aeruginosa* | Cephalosporins 4G (cefepime) | XDR *P aeruginosa* | TSA, LTF | UV | 0 qtr | NR | 0.51 | NR | NR | NR | <0.001 | NR | NR |
| Lopez-Lozano 2019^26^ | *P. aeruginosa* | Fluoroquinolones | Aminoglycosides (gentamicin) | TSA, MVA | MV | 1 | NR | 0.045 | NR | 3.01 | 0.029 to 0.061 | 0.003 | 0.857 | Prior resistance; other Abx |
| Willmann 2013^37^ | *P. aeruginosa* | Fluoroquinolones (ciprofloxacin) | Aminoglycosides (gentamicin) | TSA, LTF | UV | 1 qtr | NR | 0.27 | NR | NR | 0.003 to 0.54 | 0.048 | NR | NA |
| Willmann 2013^37^ | *P. aeruginosa* | Fluoroquinolones (ciprofloxacin) | Aminoglycosides (tobramycin) | TSA, LTF | MV | 0 qtr | NR | 0.04 | NR | NR | -0.06 to 0.14 | 0.45 | NR | Other Abx |
| Erdeljic 2011^17^ | *P. aeruginosa* | Fluoroquinolones (ciprofloxacin) | Fluoroquinolones (ciprofloxacin) | TSA | UV | 1 | RC | 0.827 | 0.349 | 2.371 | NR | 0.039 | NR | NR |
| Hocquet 2008^20^ | *P. aeruginosa* | Fluoroquinolones, antipseudomonal (ciprofloxacin) | Overproduction of MexXY-OprM^b^ | TSA, ARIMA, LTF | MV | 0, 6 | NR | 0.048 | 0.006 | NR | NR | NR | 0.811 | Prior resistance; other Abx |
| Hocquet 2008^20^ | *P. aeruginosa* | Fluoroquinolones, poorly active on P. aeruginosa (norfloxacin, ofloxacin) | Overproduction of MexXY-OprM^b^ | TSA, ARIMA, LTF | MV | 5 | NR | 0.019 | 0.004 | NR | NR | NR | 0.811 | Prior resistance; other Abx |
| Beovic 2011^16^ | *P. aeruginosa* | Penicillins [SICU] | Carbapenems | TSA, ARIMA, CCA | NR | 1 | NR | NR | NR | NR | NR | NR | NR | NR |
| Lepper 2002^25^ | *P. aeruginosa* | Penicillin/B-lact inhib (piperacillin/tazobactam) | Carbapenems (imipenem) | PCC | MV | 0 | PCC | -0.27 | NR | NR | NR | NR | NR | Other Abx; seasonal effects; patient transfers |
| Lepper 2002^25^ | *P. aeruginosa* | Penicillin/B-lact inhib (piperacillin/tazobactam) | Carbapenems (imipenem) | PCC | MV | 1 | PCC | -0.1 | NR | NR | NR | NR | NR | Other Abx; seasonal effects; patient transfers |
| Lepper 2002^25^ | *P. aeruginosa* | Penicillin/B-lact inhib (piperacillin/tazobactam) | Cephalosporins 3G (ceftazidime) | PCC | MV | 0 | PCC | 0.12 | NR | NR | NR | NR | NR | Other Abx; seasonal effects; patient transfers |
| Lepper 2002^25^ | *P. aeruginosa* | Penicillin/B-lact inhib (piperacillin/tazobactam) | Cephalosporins 3G (ceftazidime) | PCC | MV | 1 | PCC | -0.03 | NR | NR | NR | NR | NR | Other Abx; seasonal effects; patient transfers |
| Willmann 2013^37^ | *P. aeruginosa* | Penicillin/B-lact inhib (piperacillin/tazobactam) | Cephalosporins 3G (ceftazidime) | TSA, LTF (all Abx) | MV | 0 qtr | NR | 0.03 | NR | NR | -0.11 to 0.16 | 0.712 | NR | Other Abx |
| Willmann 2013^37^ | *P. aeruginosa* | Penicillin/B-lact inhib (piperacillin/tazobactam) | Cephalosporins 3G (ceftazidime) | TSA, LTF (all Abx) | MV | 0 qtr | NR | 0.02 | NR | NR | -0.13 to 0.17 | 0.82 | NR | Other Abx + cefepime |
| Lepper 2002^25^ | *P. aeruginosa* | Penicillin/B-lact inhib (piperacillin/tazobactam) | Penicillin/B-lact inhib (piperacillin/tazobactam) | PCC | MV | 0 | PCC | 0.19 | NR | NR | NR | NR | NR | Other Abx; seasonal effects; patient transfers |
| Lepper 2002^25^ | *P. aeruginosa* | Penicillin/B-lact inhib (piperacillin/tazobactam) | Penicillin/B-lact inhib (piperacillin/tazobactam) | PCC | MV | 1 | PCC | 0.01 | NR | NR | NR | NR | NR | Other Abx; seasonal effects; patient transfers |
| Aldrin 2013^14^ | *P. aeruginosa* | Penicillins extended spectrum (ampicillin/ pivampicillin; mecillinam/ pivmecillinam) | Penicillins extended spectrum (ampicillin/ pivampicillin; mecillinam/ pivmecillinam) | Poisson regr | NR | 0 | RC | 0.0104 | NR | NR | NR | <0.05 | NR | Prior resistance |
| Hocquet 2008^20^ | *P. aeruginosa* | Penicillins, antipseudomonal (piperacillin +/- tazobactam, ticarcillin +/- clavulanic acid, aztreonam) | Overproduction of MexXY-OprM^b^ | TSA, ARIMA, LTF | MV | 0, 3, 5 | NR | -0.037 | 0.029 | NR | NR | NR | 0.811 | Prior resistance; other Abx |
| Hocquet 2008^20^ | *P. aeruginosa* | Penicillins, inactive on P. aeruginosa (amoxicillin +/- clavulanic acid, cloxacillin) | Overproduction of MexXY-OprM^b^ | TSA, ARIMA, LTF | MV | 0, 1, 3 | NR | -0.015 | 0.002 | NR | NR | NR | 0.811 | Prior resistance; other Abx |
| Lepper 2002^25^ | *P. aeruginosa* | Penicillin/B-lact inhib (piperacillin/tazobactam) | Any one of imipenem, ceftazidime or piperacillin-tazobactam | TSA | MV | ≥6 | RC | NR | NR | NR | NR | NR | NR | Other Abx; seasonal effects; patient transfers |
| Baditoiu 2017^15^ | *P. aeruginosa* | Penicillin | CR *P. aeruginosa* | CCA, regr | NR | 0 qtr | NR | 0.047 | NR | NR | NR | 0.006 | 0.953 | Current+prior resistance |
| Baditoiu 2017^15^ | *P. aeruginosa* | Penicillin | CR *P. aeruginosa* | CCA, regr | NR | 1 qtr | NR | -0.0196 | 0.013 | NR | NR | 0.243 | 0.953 | Current+prior resistance |
| Baditoiu 2017^15^ | *P. aeruginosa* | Penicillin/B-lact inhib [piperacillin/tazobactam] | CR *P. aeruginosa* | CCA, regr | NR | 0 qtr | NR | 0.061 | 0.015 | NR | NR | 0.007 | 0.73 | None |
| Willmann 2013^37^ | *P. aeruginosa* | Penicillin/B-lact inhib (piperacillin/tazobactam) | 3/4 MDR^c^ *P*. *aeruginosa* | TSA, LTF | Univariate | 0 qtr | NR | 0.15 | NR | NR | NR | 0.011 | NR | NR |
| Willmann 2013^37^ | *P. aeruginosa* | Penicillin/B-lact inhib (piperacillin/tazobactam) | 3/4 MDR^c^ *P*. *aeruginosa* | TSA, LTF (all Abx) | MV | 0 qtr | NR | 0.03 | NR | NR | -0.09 to 0.15 | 0.61 | NR | Other Abx |
| Willmann 2013^37^ | *P. aeruginosa* | Penicillin/B-lact inhib (piperacillin/tazobactam) | 3/4 MDR^c^ *P*. *aeruginosa* | TSA, LTF (all Abx + cefepime) | MV | 0 qtr | NR | 0.02 | NR | NR | -0.01 to 0.14 | 0.76 | NR | Other Abx (cefepime) |
| ^a^Lag in months unless otherwise stated.  ^b^Overproduction of MexXY-OprM leads to low-level resistance to aminoglycosides, fluoroquinolones and 4G cephalosporins.  ^c^3/4 MDR *P. aeruginosa* = non-susceptibile or resistant to 3 or 4 of the following agents: piperacillin-tazobactam, ceftazidime, meropenem, ciprofloxacin. Definition as stated by Willman 2013^37^ and is not a universal definition.  **Abbreviations:** 1/2/3/4G, 1^st^/2^nd^/3^rd^/4^th^ generation; Abx, antibiotics; ARIMA, autoregressive integrated moving average; AS unit, abdominal surgery unit; B-lact inhib, B-lactamase inhibitor; BV, bivariate; CCA, Cross correlation analysis; CCC, cross-correlation coefficient; ceph, cephalosporins; CI, confidence interval; clav, clavulanic acid; coeff, coefficient; comm, community; CR, combined resistance (to ≥3 of ceftazidime, antipseudomonal penicillins, fluoroquinolones, aminoglycosides); DR, dynamic regression; HMSRRH, highest magnitude of significant response in the response horizon; hosp, hospital; ID unit, infectious disease unit; LE, link estimate; LoS, length of stay; LTF, linear transfer function; MDR, multi-drug resistant; mo, months; MV, multivariate; MVA, multivariate analysis; MVR, multivariate regression; *P. aeruginosa, Pseudomonas aeruginosa*; PCC, partial correlation coefficient; prev, prevalence; qtr, quarters; RC, regression coefficient; regr, regression; SE, standard error; SICU, surgical ICU; spp, species; TSA, time series analysis; UV, univariate; VAR, vector autoregressive model; XDR, extensively drug-resistant (resistant to at least one agent in all, or all but 1 or 2, antimicrobial categories); yr, years. | | | | | | | | | | | | | | |

**Time-lag results: *A. baumannii* and *Acinetobacter spp* (ecological study design)**

| **Study** | **Pathogen** | **Antibiotic use** | **Resistance to/ Resistance mechanism** | **Statistics** | **Model** | **Lag (mo)^a^** | **Coefficient** | | | | | | **R^2^** | **Adjusted variables** |
| --- | --- | --- | --- | --- | --- | --- | --- | --- | --- | --- | --- | --- | --- | --- |
|  |  |  |  |  |  |  | **Type** | **Coeff** | **SE** | **T-ratio** | **95% CI** | **p-value** |  |  |
| ***Acinetobacter baumannii* and *Acinetobacter spp*** | | | | | | | | | | | | | | |
| Lopez-Lozano 2019^26^ | *A. baumannii* | Carbapenems | Carbapenems | TSA | MV | 3 | NR | 0.067 | NR | 3.39 | 0.028 to 0.106 | 0.0007 | 0.859 | Prior resistance; other Abx |
| Toth 2019^33^ | *A. baumannii* | Carbapenems | Carbapenems | DR | NR | 4 | NR | 0.052 | NR | NR | 0.006 to 0.098 | 0.03 | NR | Prior resistance |
| Toth 2019^33^ | *A. baumannii* | Carbapenems | Carbapenems | VAR | BV | 1 | HMSRRH | 0.177 | NR | NR | 0.005 to 0.344 | NR | NR | Prior resistance |
| Toth 2019^33^ | *A. baumannii* | Carbapenems | Carbapenems | VAR | MV | 1-4 | HMSRRH | 0.138 | NR | NR | 0.027 to 0.275 | NR | NR | Prior resistance; other Abx |
| Lopez-Lozano 2019^26^ | *A. baumannii* | Fluoroquinolones | Carbapenems | TSA+MVA | MV | 1 | NR | 0.06 | NR | 2.89 | 0.019 to 0.100 | 0.0038 | 0.859 | Prior resistance; other Abx |
| Popovic 2020^31^ | *Acinetobacter spp* | Fluoroquinolones | Carbapenems (doripenem) | CCA | UV | 1 yr | CCC | NR | NR | NR | NR | NR | -0.78 | NR |
| ^a^Lag in months unless otherwise stated.  **Abbreviations:** *A. baumannii*, *Acinetobacter baumannii*; Abx, antibiotics; ARIMA, autoregressive integrated moving average; BV, bivariate; CCA, Cross correlation analysis; CCC, cross-correlation coefficient; CI, confidence interval; coeff, coefficient; comm, community; DR, dynamic regression; HMSRRH, highest magnitude of significant response in the response horizon; LTF, linear transfer function; mo, months; MV, multivariate; MVA, multivariate analysis; MVR, multivariate regression; qtr, quarters; RC, regression coefficient; regr, regression; SE, standard error; spp, species; TSA, time series analysis; UV, univariate; VAR, vector autoregressive model; yr, years. | | | | | | | | | | | | | | |

**Time-lag results: ESBL-producing pathogens and gram-negative bacilli (ecological study design)**

| **Study** | **Pathogen** | **Antibiotic use** | **Resistance to/ Resistance mechanism** | **Statistics** | **Model** | **Lag (mo)^a^** | **Coefficient** | | | | | | **R^2^** | **Adjusted variables** |
| --- | --- | --- | --- | --- | --- | --- | --- | --- | --- | --- | --- | --- | --- | --- |
|  |  |  |  |  |  |  | **Type** | **Coeff** | **SE** | **T-ratio** | **95% CI** | **p-value** |  |  |
| **ESBL-producers** | | | | | | | | | | | | | | |
| Vibet 2015^36^ | ESBL-producers (*E. coli, K. pneumoniae, E. cloacae*) | Carbapenems | ESBL resistance | TSA, regr | UV | 2 | LE | −0.0066 | 0.0022 | NR | NR | 0.003 | NR | None |
| Vibet 2015^36^ | ESBL-producers (*E. coli, K. pneumoniae, E. cloacae*) | Carbapenems | ESBL resistance | TSA, regr | MV | 2 | LE | −0.0028 | 0.0019 | NR | NR | 0.15 | NR | Other Abx |
| Vibet 2015^36^ | ESBL-producers (*E. coli, K. pneumoniae, E. cloacae*) | Cephalosporins 1+2G | ESBL resistance | TSA, regr | UV | 1 | LE | −0.0089 | 0.0029 | NR | NR | 0.003 | NR | None |
| Kaier 2009a^21^ | ESBL-producers (*E. coli, E. cloacae, Klebsiella, Acinetobacter, Citrobacter*) | Cephalosporins 3G | ESBL production: clav +/- 3G ceph | TSA, regr | MV | 3 | NR | 1.98 | NR | 2.5 | NR | 0.022 | 0.75 | Prior resistance; other Abx; ESBL admissions; alcohol hand rub |
| Vibet 2015^36^ | ESBL-producers (*E. coli, K. pneumoniae, E. cloacae*) | Cephalosporins 3+4G | ESBL resistance | TSA, regr | UV+MV | 5 | LE | 0.0019 | 0.0009 | NR | NR | 0.04 | NR | None (UV); other Abx (MV) |
| Aldeyab 2012^12^ | ESBL-producers | Fluoroquinolones | ESBL incidence | TSA, ARIMA | MV | 1 | NR | 0.17557 | 0.072854 | 2.41 | NR | 0.02 | 0.38 | Co-morbidity scores; other Abx (community) |
| Aldeyab 2012^12^ | ESBL-producers | Fluoroquinolone restriction (mainly ciprofloxacin) | ESBL incidence | TSA, ARIMA | MV | 2 | NR | -0.44 | NR | NR | NR | 0.017 | 0.24 | NR |
| Kaier 2009a^21^ | ESBL-producers (*E. coli, E. cloacae, Klebsiella, Acinetobacter, Citrobacter*) | Fluoroquinolones | ESBL production: clav +/- 3G ceph | TSA, regr | MV | 1 | NR | 4.43 | NR | 3.82 | NR | 0.001 | 0.75 | Prior resistance; other Abx; ESBL admissions; alcohol hand rub |
| Vibet 2015^36^ | ESBL-producers (*E. coli, K. pneumoniae, E. cloacae*) | Fluoroquinolones | ESBL resistance | TSA, regr | UV+MV | 3 | LE | 0.002 | 0.0008 | NR | NR | 0.02 | NR | None (UV); other Abx (MV) |
| Vibet 2015^36^ | ESBL-producers (*E. coli, K. pneumoniae, E. cloacae*) | Penicillins (amoxicillin) | ESBL resistance | TSA, regr | MV | 5 | LE | −0.0012 | 0.0011 | NR | NR | 0.32 | NR | Other Abx |
| Vibet 2015^36^ | ESBL-producers (*E. coli, K. pneumoniae, E. cloacae*) | Penicillins (B-lactamase resistant) | ESBL resistance | TSA, regr | UV | 2 | LE | −0.0015 | 0.0009 | NR | NR | 0.09 | NR | None |
| Vibet 2015^36^ | ESBL-producers (*E. coli, K. pneumoniae, E. cloacae*) | Penicillins (B-lactamase sensitive) | ESBL resistance | TSA, regr | UV | 1 | LE | 0.0052 | 0.004 | NR | NR | 0.2 | NR | None |
| Vibet 2015^36^ | ESBL-producers (*E. coli, K. pneumoniae, E. cloacae*) | Penicillin/B-lact inhib (amoxicillin–clavulanate) | ESBL resistance | TSA, regr | UV+MV | 3 | LE | 0.0018 | 0.0011 | NR | NR | 0.13 | NR | None (UV); other Abx (MV) |
| Vibet 2015^36^ | ESBL-producers (*E. coli, K. pneumoniae, E. cloacae*) | Penicillins +/- inhib (ticarcillin; piperacillin +/- inhib) | ESBL resistance | TSA, regr | UV | 4 | LE | −0.0052 | 0.0034 | NR | NR | 0.13 | NR | None |
| Vibet 2015^36^ | ESBL-producers (*E. coli, K. pneumoniae, E. cloacae*) | Penicillins +/- inhib (ticarcillin; piperacillin +/- inhib) | ESBL resistance | TSA, regr | MV | 4 | LE | −0.0078 | 0.0031 | NR | NR | 0.015 | NR | Other Abx |
| Lopez-Lozano 2000^27^ | Gram-negative bacilli  (*E. coli, P. aeruginosa, Proteus, Salmonella, Klebsiella, Enterobacter*, other | Cephalosporins 3G (ceftazidime) | Cephalosporins 3G (ceftazidime) | TSA, LTF | NR | 1 | NR | 0.42 | 0.096 | 4.34 | NR | <0.0001 | 0.44 | Prior resistance |
| ^a^Lag in months unless otherwise stated.  **Abbreviations:** 1/2/3/4G, 1^st^/2^nd^/3^rd^/4^th^ generation; Abx, antibiotics; ARIMA, autoregressive integrated moving average; B-lact inhib, B-lactamase inhibitor; CCA, Cross correlation analysis; ceph, cephalosporins; CI, confidence interval; clav, clavulanic acid; coeff, coefficient; comm, community; DR, dynamic regression; E. coli, Escherichia coli; ESBL, extended spectrum beta-lactamase; hosp, hospital; K. pneumoniae, Klebsiella pneumoniae; LE, link estimate; LTF, linear transfer function; mo, months; MV, multivariate; MVA, multivariate analysis; MVR, multivariate regression; P. aeruginosa, Pseudomonas aeruginosa; prev, prevalence; qtr, quarters; RC, regression coefficient; regr, regression; SE, standard error; spp, species; TSA, time series analysis; UV, univariate; VAR, vector autoregressive model; yr, years. | | | | | | | | | | | | | | |
